# Differential Response of Oral Mucosal and Gingival Cells to *Corynebacterium durum*, *Streptococcus sanguinis*, and *Porphyromonas gingivalis* Multispecies Biofilms

**DOI:** 10.3389/fcimb.2021.686479

**Published:** 2021-07-01

**Authors:** Ulrike Redanz, Sylvio Redanz, Puthalayai Treerat, Sivaraman Prakasam, Li-Jung Lin, Justin Merritt, Jens Kreth

**Affiliations:** ^1^ Department of Restorative Dentistry, School of Dentistry, Oregon Health & Science University, Portland, OR, United States; ^2^ Department of Translational Rheumatology and Immunology, Institute for Musculoskeletal Medicine, University of Münster, Münster, Germany; ^3^ Department of Periodontology, School of Dentistry, Oregon Health & Science University, Portland, OR, United States; ^4^ Department of Molecular Microbiology and Immunology, School of Medicine, Oregon Health & Science University, Portland, OR, United States

**Keywords:** oral biofilm, immune response, *Streptococcus*, *Corynebacterium*, cytokine

## Abstract

Polymicrobial interactions with oral mucosal surfaces determine the health status of the host. While a homeostatic balance provides protection from oral disease, a dysbiotic polymicrobial community promotes tissue destruction and chronic oral diseases. How polymicrobial communities transition from a homeostatic to a dysbiotic state is an understudied process. Thus, we were interested to investigate this ecological transition by focusing on biofilm communities containing high abundance commensal species and low abundance pathobionts to characterize the host-microbiome interactions occurring during oral health. To this end, a multispecies biofilm model was examined using the commensal species *Corynebacterium durum* and *Streptococcus sanguinis* and the pathobiont *Porphyromonas gingivalis*. We compared how both single and multispecies biofilms interact with different oral mucosal and gingival cell types, including the well-studied oral keratinocyte cell lines OKF4/TERT-1and hTERT TIGKs as well as human primary periodontal ligament cells. While single species biofilms of *C. durum*, *S. sanguinis*, and *P. gingivalis* are all characterized by unique cytokine responses for each species, multispecies biofilms elicited a response resembling *S. sanguinis* single species biofilms. One notable exception is the influence of *P. gingivalis* upon TNF-α and Gro-α production in hTERT TIGKs cells, which was not affected by the presence of other species. This study is also the first to examine the host response to *C. durum*. Interestingly, *C. durum* yielded no notable inflammatory responses from any of the tested host cells, suggesting it functions as a true commensal species. Conversely, *S. sanguinis* was able to induce expression and secretion of the proinflammatory cytokines IL-6 and IL-8, demonstrating a much greater inflammatory potential, despite being health associated. Our study also demonstrates the variability of host cell responses between different cell lines, highlighting the importance of developing relevant *in vitro* models to study oral microbiome-host interactions.

## Introduction

Oral mucosal surfaces are colonized by polymicrobial communities (Lamont, Koo, and Hajishengallis 2018). The polymicrobial ecology directly influences the interaction with the host mucosal surface. Normally the polymicrobial community and the host mucosal surface are in a homeostatic balance creating a mutually protective environment (Miller, Fitzsimonds, and Lamont 2019). Continued perturbations of the polymicrobial interactions with the host as well as within the polymicrobial community itself may lead to a dysbiotic state, which if not reversed, can trigger chronic inflammatory tissue destruction as seen with periodontal disease (Curtis, et al., 2020). While the polymicrobial community composition and immunopathology of dysbiosis are both well characterized and heavily investigated (Curtis, et al., 2020; Van Dyke et al., 2020), the processes responsible for maintaining symbiosis remain understudied.

The polymicrobial etiology of oral diseases also complicates the classic approaches typically employed to study microbe-host interactions. For example, monospecies cultures are unlikely to elicit a similar host response to that observed in a complex dysbiotic state. A recent study demonstrated that *Streptococcus gordonii* can interfere with *Porphyromonas gingivalis* signal transduction pathways that otherwise would regulate expression of ZEB2, an important transcriptional regulator involved in the inflammatory response of oral epithelial cells (Ohshima et al., 2019). Thus, the presence of one species can mitigate the pathogenic potential of another. Other examples include metabolic cooperativity (Miller et al., 2019) and the influence on physical properties as recently shown in a dual species model using *Streptococcus sanguinis* and *Corynebacterium durum*. Co-incubation of both species resulted in lower rates of *S. sanguinis* phagocytosis, suggesting that *C. durum* can protect *S. sanguinis* from the host innate immune response (Treerat et al., 2020). In addition, most oral microbe-host interactions occur in the context of oral biofilms, and biofilm dwelling microbes may behave quite differently from their planktonic counterparts. For example, biofilm-derived *Pseudomonas aeruginosa* evades phagocytosis by polymorphonuclear neutrophils (PMN) due to the loss of its flagellum during biofilm development (Rybtke et al., 2015). Likewise, biofilm growth stimulates natural competence development and bacteriocin production in *Streptococcus mutans* in addition to reprogramming its cellular physiology (Shanker and Federle, 2017). The biofilm matrix, which is composed of extracellular polymeric substances (EPS), provides additional physical barriers and extracellular components that interfere with the host response (Karygianni et al., 2020). This has been demonstrated for the EPS of *Bifidobacterium longum*, which has an immunomodulatory effect able to reduce the severity of chronic eosinophil-related airway disorders (Schiavi et al., 2018).

In the current study, we employed an *in vitro* polymicrobial biofilm assay that includes two highly abundant early colonizers associated with oral health, *S. sanguinis* and *C. durum*, (Mark Welch et al., 2016) in addition to a later colonizing perio-pathobiont *P. gingivalis*. This model was used to interrogate the immunological responses of different oral mucosal and gingival cell lines towards a commensal dominated multispecies biofilm.

## Materials and Methods

### Bacterial Strains, Eukaryotic Cells and Growth Conditions

The following bacterial strains were used in this study: *S. sanguinis* SK36 (Xu et al., 2007), *C. durum* JJ1 (low passage clinical isolate) (Treerat et al., 2020) and *P. gingivalis* ATCC33277 (Coykendallet al., 1980). *S. sanguinis* and *C. durum* were grown in liquid or on agar solidified Bacto™ Brain Heart Infusion medium (BHI, Becton Dickinson & Co.) at 37°C under aerobic (5% CO_2_) conditions. *P. gingivalis* was grown on Schaedler Agar with vitamin K1 and 5% sheep blood (Becton Dickinson & Co.) or in BHI medium supplemented with hemin (1 µg/ml) and menadione (5 µg/ml) in a sterile 50 ml tube (Greiner Bio-One) under anaerobic (90% N_2_, 5% CO_2_, 5% H_2_) conditions. For cultivation of *P. gingivalis*, medium and agar plates were pre-incubated under anaerobic atmosphere for at least 48 h.

Eukaryotic cell lines used in this study were: i) immortalized normal human mucosal keratinocyte cell line OKF4/TERT-1 (Dickson et al., 2000), ii) immortalized normal human gingival keratinocyte/epithelial cell line hTERT TIGKs (ATCC^®^ CRL3397™) (Moffatt-Jauregui et al., 2013) as well as iii) primary human periodontal ligament cells hPDL005.

Eukaryotic cells were grown as follows: i) OKF4/TERT-1 keratinocytes were cultured in keratinocyte serum free medium (KSFM, Gibco Invitrogen), supplemented with 0.2 ng/ml epidermal growth factor (EGF) and 25 µg/ml bovine pituitary extract (BPE) as described before (Stephen et al., 2016). ii) hTERT TIGKs were cultured in keratinocyte growth medium supplemented with hydrocortisone, epidermal growth factor, insulin, epinephrine, transferrin and bovine pituitary extract (Lonza, KGM-Gold™ Bullet kit) and glutamine 6 mM. iii) The hPDL cells used in these studies, i.e., hPDL005 were isolated from the middle third of freshly extracted pre-molar tooth of a healthy patient as per established protocols (Byoung-Moo et al., 2004; Lin et al., 2010). The isolation of hPDL cells were approved by the Oregon Health & Sciences University’s Institutional Review Board (IRB ID: STUDY00015295). The isolated hPDL cells were cultured in 100 mm tissue culture dishes in Dulbecco’s Modified Eagle Medium (DMEM), supplemented with 10% fetal bovine serum (Lin et al., 2010; Seo et al., 2004).The primary hPDL cells for performing assays were within passages 2 to 9. All cells were incubated at 37°C and 5% CO_2_ and were stimulated with bacteria at 80% confluency as described below.

### Biofilm Growth on Thermanox Discs

For scanning electron microscopy (SEM), 1 ml of bacterial culture A_600_ = 0.3 in modified chemically defined medium (CDM) (Standar et al., 2010) was added to sterile, cell-culture-treated, 13-mm Thermanox discs (ThermoFisher) housed in a 24-well plate. Thermanox discs consist of a polyolefin polymer with a hydrophilic surface and are optimized for cell attachment and growth.

After incubation at 37°C, 5% CO_2_ for 18 h, unattached cells and medium were removed and then biofilms were washed twice with Sorensen’s buffer (pH 7.2). Biofilms were fixed for 24 h at 4°C with 2% (vol/vol) glutaraldehyde in Sorensen’s buffer. Biofilms were washed once with 0.1 M sodium acetate (pH 7.2) and stored in Sorensen’s buffer until dehydration of the biofilm (ethanol gradient, processed by the Oregon Health & Science University’s Multiscale Microscopy Core). Samples were further processed using a critical point dryer (Leica CPD300) prior to sputter coating with 10 nm-thick carbon (ACE600 Coater). Imaging was completed using a Helios Nanolab 660 DualBeam SEM (FEI).

### Multispecies Biofilm Formation

**Figure 1:**
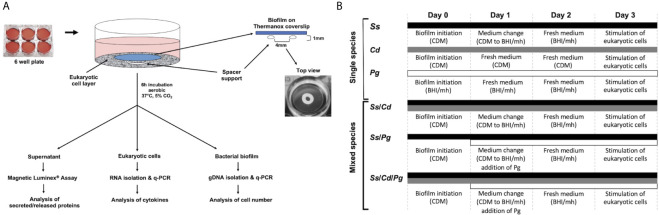
Experimental set-up for bacterial biofilm formation and its interaction with oral mucosal and gingival cells. **(A)** Oral epithelial cells were grown in 6-well plates and challenged with bacterial biofilms that were separately grown on Thermanox coverslips. Mature biofilms were developed and incubated with medium changes every 24 h for 72 h. The coverslips were carefully placed with the biofilm facing toward the epithelial cells on a spacer which forms a gap of approximately 1 mm. Subsequently, the cells were incubated for 6 h. To determine the expression of IL-6 and IL-8 with q-PCR as well as the abundance of selected immune markers with Magnetic Luminex^®^ Assay, the supernatants and the eukaryotic cells were collected and processed as described in the Materials and Methods. Biofilm cells were collected to determine the number and composition of the biofilms with q-PCR. **(B)** Thermanox cover slips were inoculated with bacteria from single species pre-cultures as described in Material and Methods at d 0. After 24 h (d 1), the growth medium was changed. For biofilms containing *P. gingivalis*, the species was added as indicated on day 1. After 48 h (d 2), the medium was changed, and at 72 h (d 3), biofilms were used for stimulating the eukaryotic cells. Different bars indicate the presence of respective species at the corresponding time points. CDM, chemical defined medium; BHI/mh, brain heart infusion supplemented with menadione (5µg/ml) and hemin (1µg/ml); *Ss*, *S. sanguinis* SK36, *Cd*, *C durum* JJ1, *Pg*, *P. gingivalis* ATCC33277.

Single, dual, and multispecies biofilms of *S. sanguinis*, *C. durum*, and *P. gingivalis* were cultivated on Thermanox coverslips (25 mm, NUNC™) as substratum (**Figure 1A**). For single species, biofilms of *S. sanguinis* and *C. durum*, overnight cultures (ONC) in BHI were centrifuged and washed twice with sterile PBS (10 min, 4,000 rpm), and each was adjusted to an optical density of A_600 _= 0.3. Next, cells were centrifuged, resuspended in the same volume of CDM (Standar et al., 2010) and seeded in 6-well plates (3 ml per well, containing 1 Thermanox coverslip). For dual species biofilms of *S. sanguinis* and *C. durum*, the bacterial density was adjusted to A_600 _= 0.3 in PBS and mixed 1:1. The bacterial suspension was subsequently centrifuged and resuspended in 1/2 volume of CDM, to reach the same cell concentration as the single species cultures, and seeded in 6-well plates as described above. Biofilms were grown under aerobic conditions at 37°C, 5% CO_2_ for 3 days with medium change every 24 h (**Figure 1B**).

For biofilms containing *P. gingivalis*, *P. gingivalis* was added to established 24 h old *S. sanguinis* single species biofilms or 24 h old 2-species biofilms (*S. sanguinis* and *C. durum*). Briefly, a 48 h-culture of *P. gingivalis* was grown anaerobically in BHI supplemented with hemin and menadione as described above, washed twice with sterile PBS and adjusted to an A_600 _= 0.3 in BHI supplemented with hemin and menadione. For *S. sanguinis*/*P. gingivalis* dual species biofilms and *S. sanguinis*/*C. durum*/*P. gingivalis* multispecies biofilms, after 24 h of biofilm growth, CDM was replaced by BHI containing *P. gingivalis* and supplemented with hemin and menadione. After 24 h of cultivation anaerobically the medium was refreshed and the biofilms were incubated for additional 24 h.

For *S. sanguinis* single species and *S. sanguinis*/*C. durum* dual species biofilms, biofilms were treated accordingly: CDM was replaced by BHI supplemented with hemin and menadione and refreshed after additional 24 h of anaerobic cultivation.

For *C. durum* single species biofilms, CDM was replaced with fresh CDM. This was done because *C. durum* single species biofilms were unable to maintain biofilm-structures when incubated in BHI (data not shown).

*P. gingivalis* single species biofilms were grown as follows: a 48 h-culture of *P. gingivalis* was washed once with sterile PBS, adjusted to A_600 _= 0.01 in BHI supplemented with hemin and menadione, seeded on Thermanox Coverslips and then incubated anaerobically for 72 h. About 3/4 of the medium was changed every 24 h. Of note, the initial optical density of *P. gingivalis* was significantly lower for single species biofilm growth compared to other species. We observed an inverse correlation between the OD and biofilm formation of *P. gingivalis*. Higher optical densities prevented *P. gingivalis* single species biofilm formation.

### Bacterial Challenge of Epithelial Cells

5x10^5^ epithelial cells were seeded in 6-well plates with the corresponding medium and incubated at 37°C, 5% CO_2_ overnight. The next day, the cells were washed gently with sterile PBS to remove unattached cells and the plates were refilled with 4 ml of fresh medium. Inoculation loops (10 µl volume, Fisherbrand Combi Loop™), serving as spacers between bacterial biofilm carrier and epithelial cells, were gently placed in the wells. Thermanox Coverslips with biofilms were gently washed once in PBS and placed in the well on top of the spacer with the biofilm side facing down. After 6 h of incubation, biofilms and spacers were removed, and the supernatants were collected at 5,000 rpm, 5 min (5810 R centrifuge, Eppendorf) and then stored at -80°C for further analyses. Cells were gently washed once with chilled PBS, lysed with 1 ml of chilled TRIzol™ (Ambion life technologies) in the cell culture dish. The lysates were then stored at -80°C for RNA isolation. As positive controls for the stimulation of eukaryotic cells, we used PMA (5 ng/ml; Sigma), *E. coli* lysates (see below) and *P. gingivalis* LPS (1 µg/ml; InvivoGen, San Diego, CA, USA).

### q-PCR Based Quantification of Bacterial Cell Numbers in Biofilms

Biofilms of *S. sanguinis*, *C. durum*, and *P. gingivalis* were grown as described above. At time point zero (just before inoculation of Thermanox Coverslips biofilm carriers), 500 µl of bacterial cell suspensions was centrifuged (5,000 rpm, 10 min, 5810 R centrifuge, Eppendorf) and cell pellets were stored at -20°C for later comparison of seeded bacterial cell numbers with final bacterial cell numbers in the mature biofilm. Mature biofilms were washed gently and transferred to a new 6-well plate containing 2 ml of sterile PBS. Biofilm cells were harvested by rigorous scratching with pipette tips, and cells were dissociated by passing through pipette tips several times. Successful biofilm removal was verified by visual inspection. The cell pellets were then collected (5,000 rpm, 10 min, 5810 R centrifuge, Eppendorf) and resuspended in 1 ml of molecular grade water (Corning). Cell suspensions were subsequently transferred into a 2 ml screw-cap tube filled with zirconia beads (BioSpec Products) and disrupted with Precellys Evolution (Bertin technologies) in 2 cycles with 8,300 rpm for 30 sec and 5 sec pause each. After every cycle, samples were placed on ice for 5 min. 1.5 µl of the extract containing gDNA was added to the mastermix (total volume 25 µl) containing 12.5 µl of PerfeCTa SYBR Green SuperMix (Quantabio), 8.5 µl of molecular grade water and 1.25 µl of 10 µM forward and reverse primers (**Table 1**) for each bacterial species. Real-time PCR was carried out with an initial incubation at 95°C for 3 min followed by 39 cycles of denaturing at 95°C for 30 sec and annealing at 60°C for 30 sec followed by amplification at 72°C for 30 sec (CFX Connect Real-Time PCR system, Bio-Rad). Bacterial cell numbers were then determined as described before (Lyons, Griffen, and Leys 2000; Sakamoto et al., 2001; Kirakodu et al., 2008). In detail, species specific genes were selected for primer design (Primer Fox). Primers were checked for potential homology to other tested species *via* BLAST-search (https://blast.ncbi.nlm.nih.gov/) and verified by PCR. Standard curves were generated as follows: targeted species-specific genes were amplified *via* standard PCR (GoTaq^®^ Green Master Mix, Promega), ligated to pGEM-T Easy (Promega) and transformed into *E. coli* DH10B competent cells. Correct insertion was verified by sequencing; sequences were identical to *P. gingivalis hmuY* (ACCESSION: CP025930), *S. sanguinis spxB* (ACCESSION: CP000387) and C. durum *periBP* (periplasmatic binding protein; ACCESSION: EKX90703). All plasmids were extracted using the Wizard plus SV minipreps DNA purification system (Promega) and linearized by cutting the plasmid backbone. The concentration (m/v) was determined by Nanodrop spectrophotometer (Thermo Scientific) and verified by electrophoresis against MassRuler™ DNA Ladder Mix (Thermo Scientific). Molecular concentrations (number of molecules per µl) were determined using the exact nucleotide sequence and the online tool DNA calculator (www.molbiotools.com). Molecular concentrations were then adjusted to 3 x 10^11^ molecules per µl, serially diluted, and analyzed *via* q-PCR. Standard curves were generated from CT-values of three biological replicates (**Figure S5**). Only these sample CT-values were assumed to be valid, which fell in the linear range of the respective standard curve. Biofilm derived gDNA was serially diluted prior to performing q-PCR to be in the linear range of the respective standard curve. CT-values were checked for linearity, and the lowest valid CT-value (corresponding to the lowest dilution) was used for cell number calculation based on the slope of the regression line of the corresponding standard curve considering all performed dilutions. Each q-PCR was performed in technical duplicates and every parameter was checked by analyzing at least three biological replicates.

**Table 1:** Oligonucleotides used for q-PCR.

Primer name	Sequence (5’ – 3’)	Reference
IL-6 F	GAAAGTGGCTATGCAGTTTGAA	(Kibea et al., 2011)
IL-6 R	GAGGTAAGCCTACACTTTCCAAGA	(Kibea et al., 2011)
IL-8 F (2)	ACTTTCAGAGACAGCAGAGC	this study
IL-8 R (2)	ACAGAGCTGCAGAAATCAGG	this study
GAPDH F (2)	CAAAAGGGTCATCATCTCTGC	this study
GAPDH R ( 2)	GTTGTCATGGATGACCTTGG	this study
qPCR_SK36_spec_SpxB_F	TAAATTCGGCGGCTCAATCG	this study
qPCR_SK36_spec_SpxB_R	GCGATACCGTTGTACATTGG	this study
qPCR_Pg_spec_hmuy_F	CGATTTGAACTGGGACATGG	this study
qPCR_Pg_spec_hmuy_R	TCCATCTGATGACCATCAGG	this study
Cd_spec_periBP_qPCR_F	CATGTTCACCAAGGATGAGG	this study
Cd_spec_periBP_qPCR_R	AGATCAAGTGCTTGGTCACC	this study

### Determination of Chemokine mRNA Expression by RT-q-PCR

After 6 h stimulation, the monolayer of the cells was washed with chilled PBS and the RNA was isolated and cDNA was generated as described previously (Chomczynski and Mackey, 1995). IL-6 and IL-8 gene expression was analyzed using SYBR Green based real-time PCR (qRT-PCR) (PerfeCTa SYBRgreen supermix, Quanta bio), in a Bio-Rad CFX Connect real-time PCR system. The experiments were performed in technical duplicates. Each data point represents the average of at least three independent experiments. Gene expression was normalized to the housekeeping gene glyceraldehyde-3-phosphate dehydrogenase (GAPDH) according to the 2^-ΔΔCT^ method (Livak and Schmittgen, 2001). A non-template control (NTC) was included in each qRT-PCR run. All qPCR reactions were performed under the following conditions: initial incubation at 95°C for 3 min followed by 39 cycles of denaturing at 95°C for 30 sec and annealing at 58°C for 30 sec followed by amplification at 72°C for 30 sec.

### Quantification of Cytokine and Chemokine Release

The quantification of cytokines in the supernatant of 6 h stimulated immortalized normal human gingival keratinocyte cell line hTERT TIGKs (ATCC^®^ CRL3397™) was determined by the Magnetic Luminex^®^ Assay (human premixed multi-analyte kit, R&D systems) according to manufacturer’s instructions. The following cytokines were tested: IL-1 β, CXCL8/IL-8, TNF-α, IL-6, IL-10 and CXCL1/GRO-α. The cytokines were measured as duplicates on a Luminex^®^200 System. Standard curves and concentrations were calculated by using xPONENT Software v3.1 (Luminex Corporation).

### *E. coli* Lysate Preparation as Immune Stimulant

*E. coli* (DH5B10) lysates were prepared as follows. A stationary phase culture of *E. coli* was diluted 1:40 in a total volume of 25 ml fresh BHI and grown in a 50 ml flask. Cells were cultivated at 37°C with agitation at 200 rpm until reached the mid-exponential phase of growth (A_600_ = 0.6-0.8). Bacterial cells (1 ml aliquot) were pelleted at 13,000 rpm for 2 min (5424 centrifuge, Eppendorf) and then washed once with an equal volume of sterile PBS. Subsequently, bacterial cells were resuspended in 500 µl of sterile PBS and boiled at 99°C for 10 min. *E. coli* lysate was then collected by centrifugation at 13,000 rpm for 2 min and stored at -20°C until further analyses.

### Crystal Violet Staining

Biofilm formation was determined by crystal violet staining as described previously (Redanz et al., 2012)

### Statistical Analysis

All experiments were performed at least three biological replicates. Statistical significance was determined by students *t*-test using the data analysis tool GraphPad Prism version 8.0. P values less than 0.05 were considered statistically significant.

## Results

### Development of *S. sanguinis*, *C. durum*, and *P. gingivalis* Single, Dual, and Multispecies Biofilms

**Figure 2:**
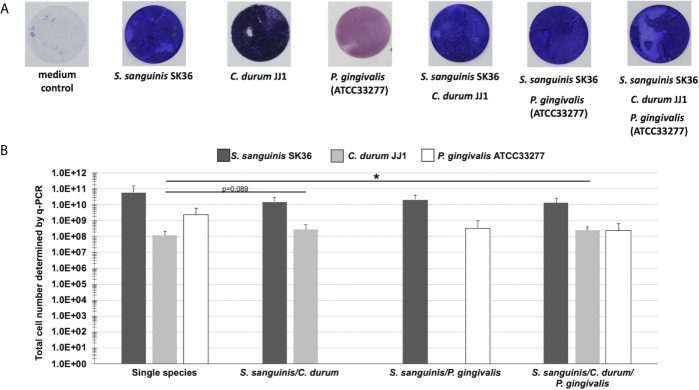
Biofilm growth of single, dual, and multispecies of *S. sanguinis*, *C. durum*, and *P. gingivalis* on Thermanox coverslips. **(A)** Bacterial biomass of biofilms grown on Thermanox coverslips for 3 days was visualized with crystal violet staining. A Thermanox coverslip incubated in medium only served as the negative control. Pictures are representative of 3 biological replicates. **(B)** Determination of total cell numbers using q-PCR. Biofilms grown on Thermanox coverslips were removed and bacterial DNA was isolated. The number of bacterial cells was calculated based upon standard curves of serially diluted DNA. Data represent the averages and standard deviations of 3 biological replicates. (p-value indicated). *p < 0.05 (Students t-test, two-tailed, paired).

A detailed analysis of the biogeographical distribution of supragingival plaque bacteria reported a high abundance and close association of *Corynebacterium* and *Streptococcus* species in the biofilm community (Mark Welch et al., 2016). *Porphyromonas*, although not as abundant, seems to co-localize with *Streptococcus* cells (Mark Welch et al., 2016). Here, we used the reference strains *S. sanguinis* SK36, *P. gingivalis* ATCC33277, and a low passage clinical isolate of *C. durum* JJ1 to develop a robust model of single, dual, and multispecies *in vitro* biofilms reflecting the close association of the three species in supragingival plaque. Biofilms were cultivated on Thermanox discs for 72 h (**Figure 1A**) with daily replacement of the growth medium (**Figure 1B**). Crystal violet staining of the developed biofilms revealed readily attached cells with visible biomass build-up for all single and multispecies biofilms (**Figure 2A**).

Using qPCR, we determined the total cell number to validate the presence of individual bacterial species in the multispecies biofilms. Consistent with the crystal violet staining results, the single species biofilm controls all amassed a considerable number of cells attached to the substratum (**Figure 2B**), with *S. sanguinis* reaching 6.01x10^10^ CFU, followed by *P. gingivalis* (2.45x10^9^ CFU), and *C. durum* (1.19x10^8^ CFU). The dual species biofilms of *S. sanguinis*/*C. durum* and *S. sanguinis*/*P. gingivalis* exhibited a numerically dominant distribution of *S. sanguinis* (1.5x10^10^/2.07x10^10^ CFU) over the two other species (2.78x10^8^ CFU for *C. durum* and 3.4x10^8^ CFU for *P. gingivalis*). A similar result was determined for the multispecies biofilms, with 1.34x10^10^, 2.43x10^8^, and 2.53x10^8^ CFU of *S. sanguinis*, *C. durum*, and *P. gingivalis*, respectively.

### Proportional and Structural Analysis of the Mature Biofilms

**Figure 3:**
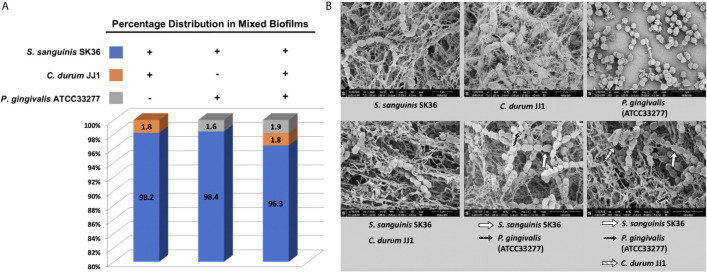
Compositional analysis and ultrastructure of mature biofilms. **(A)** The composition of dual and multispecies biofilms is presented as a percentage of individual species based on the total number of bacteria determined by q-PCR. **(B)** The ultrastructure of mono, dual, and multispecies biofilms was visualized with scanning electron microscopy (SEM). Higher magnification pictures (x35,000) are presented to depict the individual species morphologies in the biofilm. Individual species are indicated by arrows. Additional images of representative biofilms with lower magnification are given in the supplemental material section (**Figure S3**).

The percentage distribution was calculated for the dual and multispecies biofilms (**Figure 3A**). In all three cases, *S. sanguinis* was the dominant species at 98.2% (*S. sanguinis*/*C. durum*), 98.4% (*S. sanguinis*/*P. gingivalis*), and 96.3% in the three species biofilm. *C. durum* accounted for 1.8% of the total in the dual species biofilm with *S. sanguinis* and the multispecies biofilm. *P. gingivalis* slightly increased from 1.6% of the total in the *S. sanguinis* dual species biofilm to 1.9% in the three species biofilm.

The biofilm structure and bacterial morphology were evaluated using SEM (**Figures 3B** and **S3**). The significant difference between the three single species biofilms was the amount of extracellular matrix material associated with the biofilms. *S. sanguinis* and *C. durum* showed extensive production of web-like matrix material that enmeshed the cells often completely whereas *P. gingivalis* produced far less matrix material. Interestingly, all multispecies biofilms produced thicker matrix materials, which likely reflected the dominant presence of *S. sanguinis*. We were also able to identify the members of each bacterial species in the micrographs with the exception of the *S. sanguinis*/*C. durum* dual species biofilm, (**Figure 3B**; depicted by arrows). In summary, the multispecies biofilm model shows the hallmark of biofilm development with the presence of extracellular matrix material and all species present, albeit in different proportions with *S. sanguinis* numerically dominating the community.

### IL-6 and IL-8 Gene Expression Response of Different Mucosal and Gingival Cell Lines to Bacterial Biofilms

Our aim was to challenge distinct mucosal and gingival cell lines with a biofilm comprised of relevant commensal and pathobiont species. Since our model yielded a consistently high proportion of *S. sanguinis*, an abundant species in early dental biofilms (Kreth et al., 2017), our model was likely to reflect the early stages of oral biofilm development. To determine how epithelial cells responded to such biofilms, *in vitro* biofilms grown on Thermanox slides were placed on OKF4/TERT-1, hTERT TIGKs, and human primary periodontal ligament cells (hPDL005). Direct contact between the bacterial biofilm and the oral epithelial cell lines was prevented by the ring-shaped spacer, which, as a result, would position the biofilm about 1 mm over the epithelial cells (**Figure 1A**). We initially determined the inflammatory response after 6 h of biofilm exposure by measuring the expression of the IL-6 and IL-8 (CXCL8) genes.

**Figure 4:**
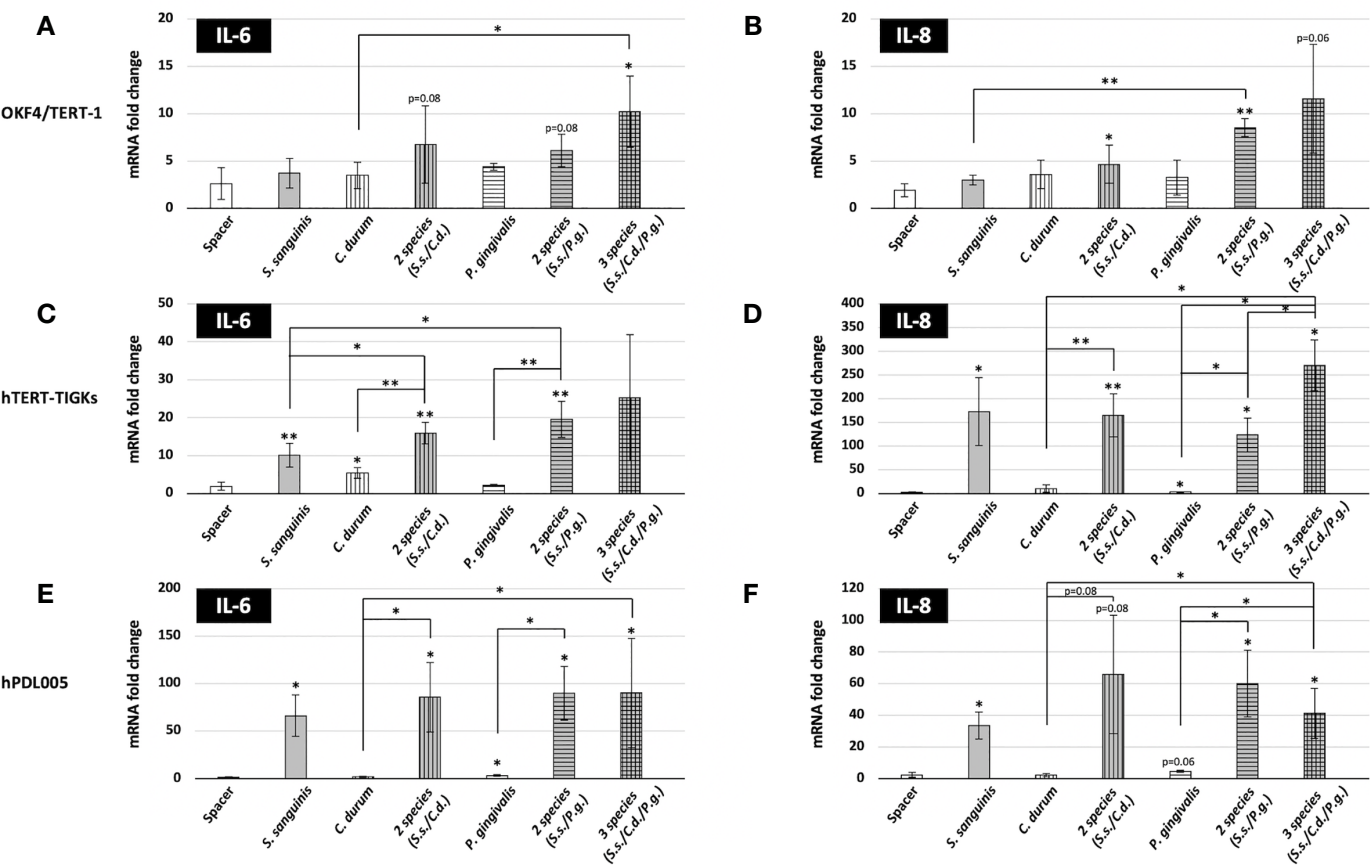
Expression of IL-6 and IL-8 genes in oral mucosal and gingival cells after biofilm challenge. Gene expression was assessed after a 6-h challenge with biofilms. The coverslips were placed with the biofilms facing the eukaryotic cells, but separated by the spacer as shown in **Figure 1** and then incubated at 37 °C with 5% CO_2_. After biofilm challenge, host cells were removed, RNA was isolated, and the expression of IL-6 and IL-8 genes was determined by qRT-PCR. Data represent the averages and standard deviations of at least 3 biological replicates. **(A, B)** OKF4/TERT-1; **(C, D)** hTERT-TIGKS; **(E, F)** hPDL005. (p-value indicated) *P ≤ 0.05; **P ≤ 0.01.

As presented in **Figures 4A–F**, different cell types exhibited different gene expression in response to bacterial biofilms. OKF4/TERT-1 cell yielded a moderate response to the biofilms, showing slight expression changes in IL-6 and IL-8 gene expression. This cell line also exhibited a weak response to established immunostimulants, including PMA, *P. gingivalis* LPS and *E. coli* lysate (**Figure S4**). In contrast, both hTERT TIGKs and hPDL005 cells were substantially responsive based on a dramatic increase in IL-6 and IL-8 gene expression as well as differential responses to the single and multispecies biofilms. IL-8 gene expression was the strongest in the hTERT TIGKs samples while both IL-6 and IL-8 expression patterns were only marginal difference in the hPDL005 cells. Variability was also higher for hPDL005 samples, perhaps because these are primary cells.

When comparing the mono, dual, and multispecies biofilms, distinct inflammatory responses were evident. Surprisingly, *S. sanguinis*, considered a benign if not beneficial bacterium, was able to significantly induce both IL-6 (10-fold in hTERT TIGKs; 66-fold in hPDL005) and IL-8 (173-fold in hTERT TIGKs; 33-fold in hPDL005) gene expression. However, periodontitis associated *P. gingivalis* (Hajishengallis et al., 2011) and less characterized *C. durum* (Treerat et al., 2020) showed minimal induction. Counterintuitively, both seemed to either prevent the induction of IL-6 and IL-8 expression in hTERT TIGKs and hPDL005 cells or simply failed to activate these cells. The dual and multispecies biofilms were able to induce IL-6 and IL-8 expression to a comparable extent as *S. sanguinis* alone, suggesting that the numerical abundance of *S. sanguinis* in the mixed biofilms was the dominant factor.

Although we were mainly interested in the early response of the mucosal and gingival cells, we also challenged hTERT TIGKs cells with *P. gingivalis* and *C. durum* biofilms for 24 h. We were not able to include *S. sanguinis* in these late response experiments due to its rapid growth in the cell culture medium resulting in hTERT TIGKs cytotoxicity. As shown in **Figure S1**, no significant difference was observed in the IL-6 and IL-8 expression of hTERT TIGKs cells when comparing both time points of bacterial challenges. However, a significant difference was observed in IL-6 expression when the cells were stimulated by *E. coli* lysate, showing an early induction of IL-6.

Overall, the results suggest that these mucosal and gingival cells can distinctively respond to different bacterial species in the biofilms, resulting in differential inflammatory responses.

### Production and Secretion of Key Immune Markers After Bacterial Challenge

**Figure 5:**
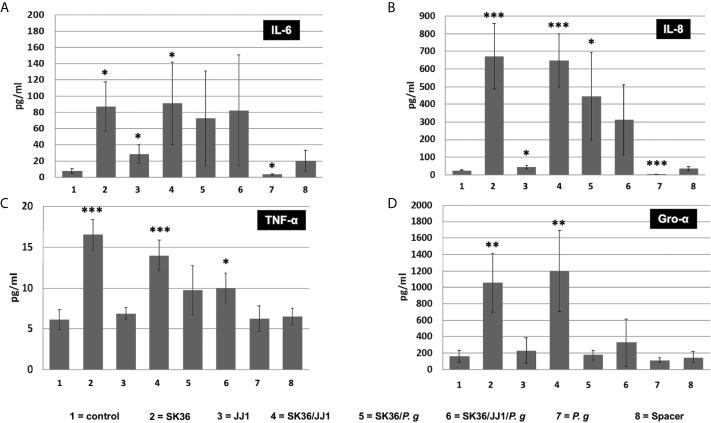
. Cytokine response of immortalized human gingival keratinocyte cell line hTERT-TIGKs. Supernatants were collected 6 h after biofilm challenge. The protein abundance (pg/ml) of CXCL8/IL-8, TNF-α, IL-6, and CXCL1/GRO-α was determined using a Magnetic Luminex Assay. Unchallenged cells (no spacer, “control”) and the spacer placed onto the confluent cell layer (“spacer”) served as controls. **(A)** IL-6; **(B)** IL-8; **(C)** TNF-α; **(D)** Gro-α. Data represent the averages and standard deviations of at least 3 biological replicates. Statistical differences are shown as comparison to the no spacer-control (1=column). *P ≤ 0.05; **P ≤ 0.01; ***P ≤ 0.001.

We further investigated if increasing IL-6 and IL-8 expression could contribute to an increase in secreted cytokines in hTERT TIGKs cells. After 6 h of bacterial biofilm challenge, both IL-6 and IL-8 levels in the supernatants were correlated with their gene expressions (**Figures 5A, B**). In addition, the presence of *S. sanguinis* in the dual and multispecies biofilms exhibited the similarly dominant effect on other species.

An analogous result was found in TNF-*α* and Gro-α levels with a key difference (**Figures 5C, D**). The dual and multispecies biofilms of *S. sanguinis*/*P. gingivalis* and *S. sanguinis*/*C. durum*/*P. gingivalis* were unable to induce TNF-α and Gro-α production to a similar degree as the single *S. sanguinis* biofilms and the dual species biofilm with *C. durum*. While *S. sanguinis* and *S. sanguinis/C. durum* biofilms could induce a 10-fold increase in secreted Gro-α compared to the control, no significant difference was detected in other biofilms. Furthermore, Gro-α levels were significantly lower in the three species biofilm compared to the *S. sanguinis*/*C. durum* biofilm, suggesting that the presence of *P. gingivalis* specifically inhibited Gro-α production. We also measured the production of other key cytokines, including IL-1β and IL-10, in the supernatants; however, neither of them showed statistically significant difference (**Figures S2A, B**).

## Discussion

The human mucosae is in constant contact with the microbiome (Groeger and Meyle, 2019). Consequently, a homeostatic relationship between the host and microbial community is crucial to maintain health. Continued imbalances can lead to dysbiosis and chronic infections (Frias-Lopez and Duran-Pinedo, 2020). While the immunology and microbiology of periodontitis is well characterized, far less is known about molecular mechanisms supporting oral health. To approach this question, we wanted to develop a stable biofilm model that is dominated by commensal bacteria, but also contains a pathobiont to determine whether its presence influences the immunological response to the consortium. Our model included both *S. sanguinis* and *C. durum*, which are both abundant members of commensal biofilms, are associated with oral health, and exhibit metabolic communication *in vitro* (Stingu et al., 2008; Chen et al., 2018; Zhu et al., 2018; Schoilew et al., 2019; Treerat et al., 2020). Further, biogeographical analysis demonstrated *in situ* co-aggregation between *Corynebacterium* and *Streptococcus*, although the specific species have yet to be determined (Mark Welch et al., 2016). It is also worth noting that *S. sanguinis* has been found to be one of the most transcriptionally active species in healthy dental plaque, which is further evidence for its numerical abundance in commensal biofilms (Peterson et al., 2014).

While the ecological roles of oral streptococci have been investigated in great detail (Kreth et al., 2017; Abranches et al., 2018), little is known about oral corynebacteria. However, clinical studies indicate they likely play significant roles in the development of the dental biofilm (Ferrer and Mira, 2016; Esberg et al., 2020; Diaz and Valm, 2020). The role of *P. gingivalis* in periodontal disease is well documented (How, Song, and Chan 2016; Mulhall, Huck, and Amar 2020). Consistent with its potential to initiate the development of periodontal disease as a low abundant pathobiont (Hajishengallis et al., 2011; Hajishengallis, 2014), *P. gingivalis* comprised <2% of the total population in our multispecies biofilms. Overall, *P. gingivalis* abundance in our model is consistent with other reports of multispecies biofilm models used to investigate host-immune responses (Brown et al., 2019) as well as saliva-derived biofilms (Buskermolen et al., 2018).

We decided to follow the early response of the selected mucosal and gingival epithelial cells to a controlled bacterial biofilm challenge. We deliberately avoided direct biofilm cell contact by the use of a spacer to prevent any mechanical disruption of the cell-layers. In addition, we chose this approach, (i) to mimic the crevice fluid flow (Goodson, 2003) and (ii) to provide eukaryotic cells with sufficient amount of nutrition and oxygen. We observed that direct contact over the given time period resulted in cell death (data not shown). As expected, the placement of the spacer did not elicit a significant response for the tested cytokines (**Figures 4** and **5**). Compared to the other cell lines, OKF4/TERT-1 showed a surprisingly weak response to positive control immune stimulants like PMA or LPS. Potentially, this resulted from an underrepresentation of available LPS-binding protein since the standard cultivation medium fir OKF4 does not contain any serum. However, our data were similar to previous results with this cell line. For example, IL-8 expression has been examined after a six-hour challenge with single species biofilms and planktonic cells of *S. sanguinis* and *P. gingivalis* (albeit different strains). OKF4/TERT-1 challenged with both species showed a comparable low expression of IL-8 mRNA relative to our data (Peyyala et al., 2011). Moreover, IL-6 levels in supernatants also seemed to be low for *S. sanguinis* and *P. gingivalis* (Peyyala et al., 2012). Both of these studies were performed under anaerobic conditions, while ours was performed aerobically in the presence of 5% CO_2_, suggesting that oxygen does not have an obvious effect upon the production of the cytokines examined in our study. Currently, it is unclear why OKF4 is not mounting a robust IL-6 and IL-8 response, as both cytokines play a central role in the inflammatory response to microorganisms (Bickel, 1993; Tanaka et al., 2014). OKF4 cells were isolated from the floor of the mouth, where they would presumably be in constant contact with bacteria. It is quite possible that the original anatomic *in situ* location renders the cells inert to a bacterial challenge avoiding an unnecessary chronic inflammatory response. It is also possible that the here tested time frame of 6 hours might not be long enough to exert a robust response. In the related cell line OKF6, also an isolate from the floor of the mouth, a 24-hour challenge of live biofilms that included *P. gingivalis* and commensal *Streptococcus mitis* showed a significant increase in IL-6 and IL-8 expression (Ramage et al., 2017). Interestingly, OKF4 and OKF6 were isolated from males of different age [OKF4 28 years old *vs* OKF6 57 years old (Dickson et al., 2000)] and age-related differences in the response that potentially alter innate immunity are plausible (Shaw et al., 2013), but that has not been tested. Although the biofilms grown for this study seemed to be in general quite stable, it is conceivable that bacterial cells are released by the mature biofilms. An extended co-culturing period would likely increase the amount of released bacterial cells potentially triggering a differential immunological response. This hypothesis could explain the differences between the here observed weak response of OKF4 cells (after 6 hours) and the more robust response after 24 hours (Ramage et al., 2017). It is also possible that the modest responsiveness of OKF4 could simply be a consequence of its conversion to an immortalized cell line (Alge et al., 2006).

A more pronounced response in the expression of IL-6 and IL-8 was observed with the hTERT-TIGKs cell line and primary hPDL005 cells. This was most obvious for *S. sanguinis* either alone or in combination with the other bacterial species, suggesting that *S. sanguinis* has a dominant effect on stimulating the tested cytokines. IL-6 expression was highest in the primary periodontal ligament cells, whereas IL-8 exhibited the greatest fold-increase in the hTERT-TIGKs cell line. Surprisingly, both *C. durum* and *P. gingivalis* did not elicit a noticeable response in either of these cell types.

The different immunological responses of the here investigated oral derived eukaryotic cells was unexpected. However, the relative anatomical site of isolation in relation to the encounter of biofilms or planktonic cells could explain the responses. Periodontal ligament cells (hPDL005 cells) are fibroblast-like cells with immunological function connecting the tooth root to the alveolar bone and are most-likely in close proximity to biofilms (Jonsson et al., 2011). hTERT-TIGKs (Telomerase Immortalized Gingival Keratinocytes) originated from gingival epithelium (Moffatt-Jauregui et al., 2013) and are therefore also in close proximity to biofilms. OKF4 as well as OKF6, as mentioned before, were isolated from the floor of the mouth and might primarily encounter planktonic cells, thus the anatomical site could dictate the individual responses to biofilm or planktonic cells. Subsequent experiments will have to test this hypothesis and clarify if different oral keratinocytes/epithelial cells respond selectively more robust to planktonic or biofilm bacteria.

The cellular response to *S. sanguinis* was further confirmed by measuring the abundance of these cytokines in the culture media of the hTERT-TIGKs cell line. A significant increase in secreted IL-6, IL-8, TNF-α, Gro-α and IL-1β was measured, but the amount of secreted IL-1β as well as IL-10 was modest when compared to the control. Similar results were also observed when *S. sanguinis* was grown in dual species biofilms with *C. durum*. Again, *C. durum* and *P. gingivalis* single species biofilms did not show a difference in the measured cytokine levels confirming our gene expression results. There was, however, an obvious difference between *C. durum* and *P. gingivalis* and their ability to influence the effect of *S. sanguinis* on TNF-α and Gro-α. The amount of both cytokines was significantly reduced when *P. gingivalis* was present in dual and multispecies biofilms, while *C. durum* was not able to influence the effect of *S. sanguinis*. Our results raise an interesting question: is *C. durum* not recognized by the tested cell types, or does it actively suppress an immunological response as has been shown for *P. gingivalis* (Hajishengallis and Diaz, 2020)? Based upon our results, it appears as if there is either no active immunosuppressive mechanism employed by *C. durum* or this ability is regulated, since it would otherwise have been expected to function in each of the multispecies biofilm conditions. *C. durum* has not been investigated for its ability to modulate the immune response, but it has been shown to increase the longevity of *Caenorhabditis elegans via* the secretion of monoamines and *N*-acetyl monoamines (Kim et al., 2020), suggesting that this species can influence the health of the host, as would be expected from a true commensal organism. We also previously demonstrated how *S. sanguinis* phagocytosis is inhibited by coculture with *C. durum* (Treerat et al., 2020). The interactions of commensal species and their potentially coordinated influence on oral epithelial responses toward pathogenic species is certainly more complex. It seems that oral streptococci, as shown with *S. gordonii* have the ability to antagonize the effect of pathogenic species like *P. gingivalis* on oral epithelial responses by modulating the expression of several genes, including genes involved in cell cycling (Mans et al., 2009). Thus, the response will have a much broader impact on mucosal cell physiology and it is certainly warranted to further explore the mutual relationship of both species in the context of oral health.

Although *S. sanguinis* biofilms have been demonstrated to be a poor stimulant of pro-inflammatory cytokines in OKF-4 (Peyyala et al., 2012), which has also been confirmed with cell wall extracts that failed to induce a significant upregulation of IL-8 in the gingival keratinocyte cell line Ca9-22 (Peyret-Lacombe et al., 2009), we present a different picture here. It seems that *S. sanguinis* single and multispecies biofilms are able to induce significant proinflammatory responses in hTERT-TIGKs and primary periodontal ligament cells. The proinflammatory ability of *S. sanguinis* has been demonstrated with peripheral blood monocytes (Kjeldsen et al., 1995) as well as platelets (McNicol et al., 2011; Cognasse et al., 2014). This is possible linked to the secretion of a CD14-binding protein which stimulates cytokine synthesis (Banks et al., 2002). Our observation is in line with *S. sanguinis* extraoral role as important etiologic agent of infective endocarditis (Baker, Nulton, and Kitten 2019), which is a biofilm associated disease (Zhu et al., 2018). Furthermore, it is important to consider that *S. sanguinis* exhibited robust growth in the host cell growth medium, potentially influencing the cellular response due to its metabolic products (such as H_2_O_2_ or lactic acid) that are released into the medium. These factors should be taken into consideration for future work.

In conclusion, our study reveals how oral bacteria associated with oral health interact with oral mucosal and gingival cells. The differential responses towards commensal species in our study of *S. sanguinis* and *C. durum* will be further examined in future studies, since both commensals appear to have vastly different inflammatory potentials. Unlike *S. sanguinis*, *C. durum* exhibits a surprisingly low inflammatory stimulation that may be crucial for maintaining the homeostatic relationship between the oral microbiome and host.

## Data Availability Statement

The datasets presented in this study can be found in online repositories. The names of the repository/repositories and accession number(s) can be found below: NCBI; accession numbers: P. gingivalis hmuY (ACCESSION: CP025930), S. sanguinis spxB (ACCESSION: CP000387) and C. durum periBP (periplasmatic binding protein; ACCESSION: EKX90703).

## Author Contributions

UR planned and performed experiments, wrote and revised the manuscript. SR planned experiments, wrote and revised the manuscript. PT planned and performed experiments, and revised the manuscript. SP planned experiments, and revised the manuscript. L-JL performed experiments. JM provided intellectual input, wrote and revised the manuscript. JK provided intellectual input, planned experiments wrote and revised the manuscript. All authors contributed to the article and approved the submitted version.

## Funding

This project was supported by NIH grants DE022083, DE023850, and DE028252 to JM and NIH grants DE029612, DE021726 and DE029492 to JK. Electron microscopy was performed at the Multiscale Microscopy Core (MMC) with technical support from the Oregon Health & Science University (OHSU)-FEI Living Lab and the OHSU Center for Spatial Systems Biomedicine (OCSSB). We also like to thank Dorian LaTocha from the OHSU Flow Cytometry Shared Resource (FSCR) Core for technical help with the Luminex^®^ Assay.

## Conflict of Interest

The authors declare that the research was conducted in the absence of any commercial or financial relationships that could be construed as a potential conflict of interest.
