# Supplementary material for: Differential Response of Oral Mucosal and Gingival Cells to Corynebacterium durum, Streptococcus sanguinis, and Porphyromonas gingivalis Multispecies Biofilms
Source: Front Cell Infect Microbiol. 2021 Jul 1;11:686479. doi: 10.3389/fcimb.2021.686479 (PMC8282179; doi:10.3389/fcimb.2021.686479)
Supplement: Supplementary file 1 [file Presentation_1.pptx]

## Slide 1
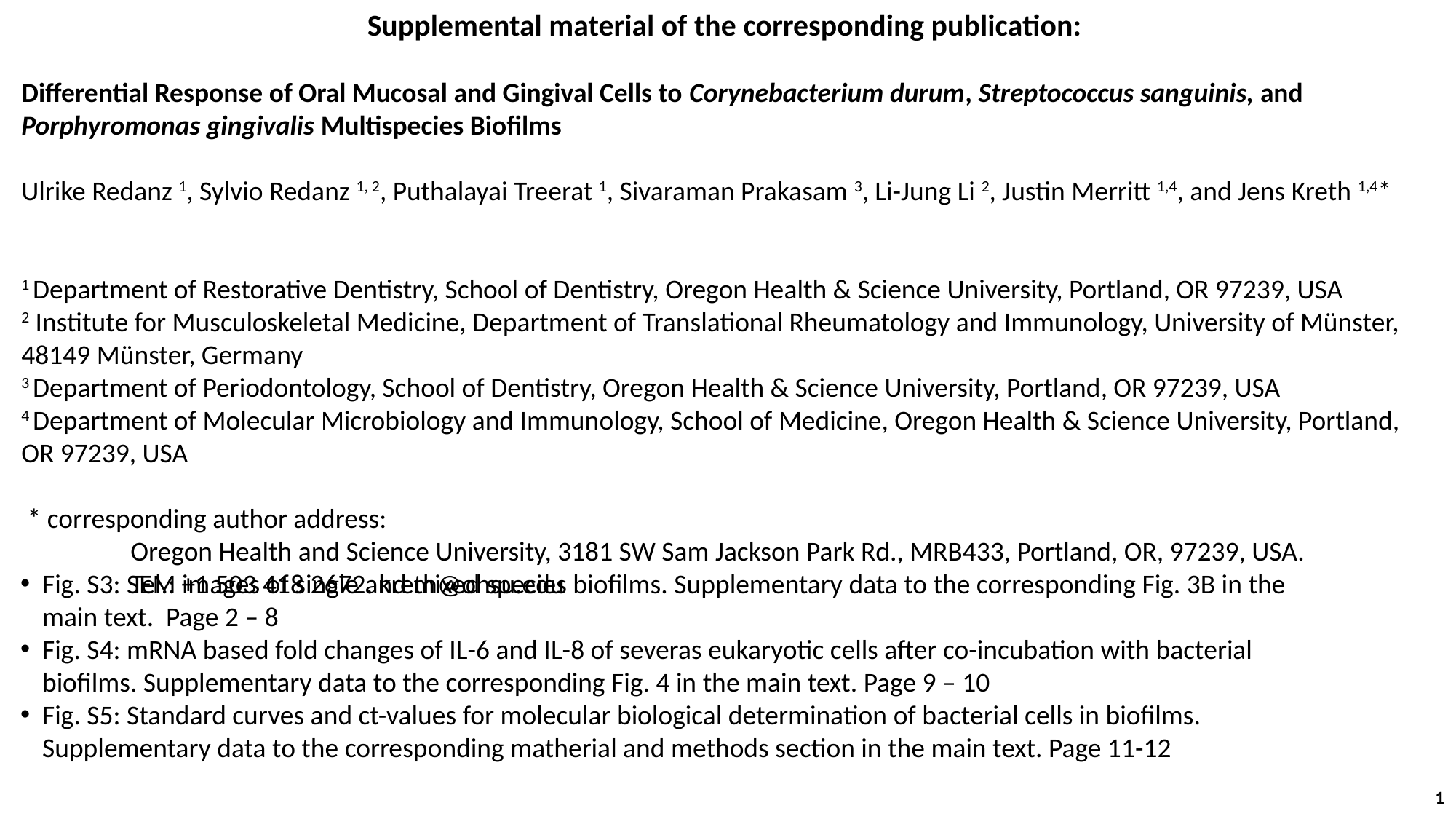

Supplemental material of the corresponding publication:
Differential Response of Oral Mucosal and Gingival Cells to Corynebacterium durum, Streptococcus sanguinis, and Porphyromonas gingivalis Multispecies Biofilms
Ulrike Redanz 1, Sylvio Redanz 1, 2, Puthalayai Treerat 1, Sivaraman Prakasam 3, Li-Jung Li 2, Justin Merritt 1,4, and Jens Kreth 1,4*
1 Department of Restorative Dentistry, School of Dentistry, Oregon Health & Science University, Portland, OR 97239, USA
2 Institute for Musculoskeletal Medicine, Department of Translational Rheumatology and Immunology, University of Münster, 48149 Münster, Germany
3 Department of Periodontology, School of Dentistry, Oregon Health & Science University, Portland, OR 97239, USA
4 Department of Molecular Microbiology and Immunology, School of Medicine, Oregon Health & Science University, Portland, OR 97239, USA
 * corresponding author address:
	Oregon Health and Science University, 3181 SW Sam Jackson Park Rd., MRB433, Portland, OR, 97239, USA.
	Tel.: +1 503 418 2672. kreth@ohsu.edu
Fig. S3: SEM images of single and mixed species biofilms. Supplementary data to the corresponding Fig. 3B in the main text. Page 2 – 8
Fig. S4: mRNA based fold changes of IL-6 and IL-8 of severas eukaryotic cells after co-incubation with bacterial biofilms. Supplementary data to the corresponding Fig. 4 in the main text. Page 9 – 10
Fig. S5: Standard curves and ct-values for molecular biological determination of bacterial cells in biofilms. Supplementary data to the corresponding matherial and methods section in the main text. Page 11-12
1

## Slide 2
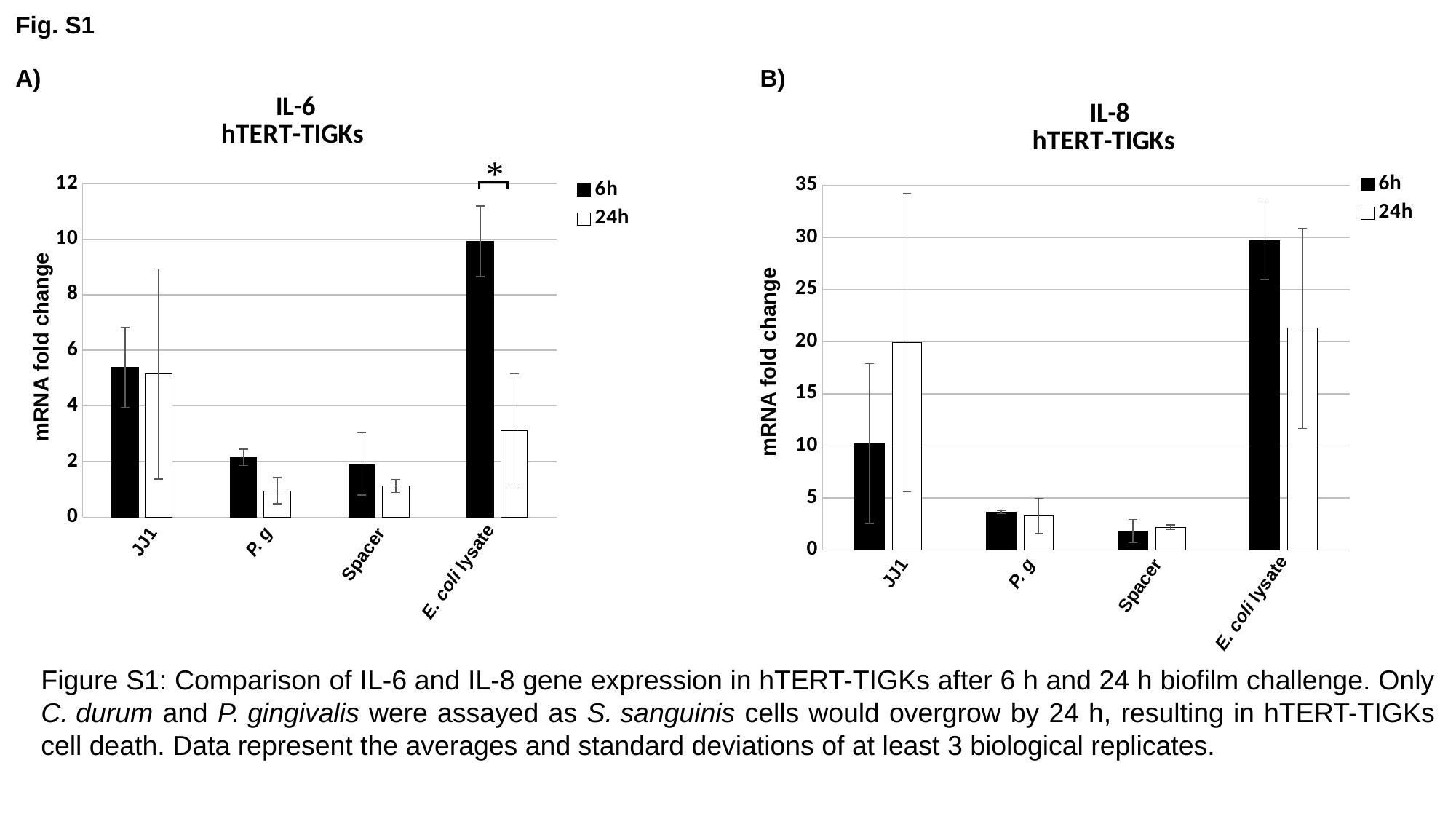

Fig. S1
A)
B)
### Chart: IL-8
hTERT-TIGKs
| Category | | |
|---|---|---|
| C.durum | 10.206668027978788 | 19.903201995051287 |
| P.g.ATCC33277 | 3.668320651437203 | 3.2637157330089472 |
| LW spacer fixed | 1.8233053523536438 | 2.2052958558110376 |
| LPS (E. coli lysat ) | 29.68437928700287 | 21.27723312841719 |mRNA fold change
### Chart: IL-6
hTERT-TIGKs
| Category | | |
|---|---|---|
| C.durum | 5.3941187424303605 | 5.14823815044464 |
| P.g.ATCC33277 | 2.154529883395058 | 0.9508189382921776 |
| LW spacer fixed | 1.9112835574573512 | 1.1173866164550152 |
| LPS (E. coli lysat ) | 9.924839235697327 | 3.107349066728243 |
mRNA fold change
JJ1
P. g
Spacer
JJ1
E. coli lysate
P. g
Spacer
E. coli lysate
Figure S1: Comparison of IL-6 and IL-8 gene expression in hTERT-TIGKs after 6 h and 24 h biofilm challenge. Only C. durum and P. gingivalis were assayed as S. sanguinis cells would overgrow by 24 h, resulting in hTERT-TIGKs cell death. Data represent the averages and standard deviations of at least 3 biological replicates.

## Slide 3
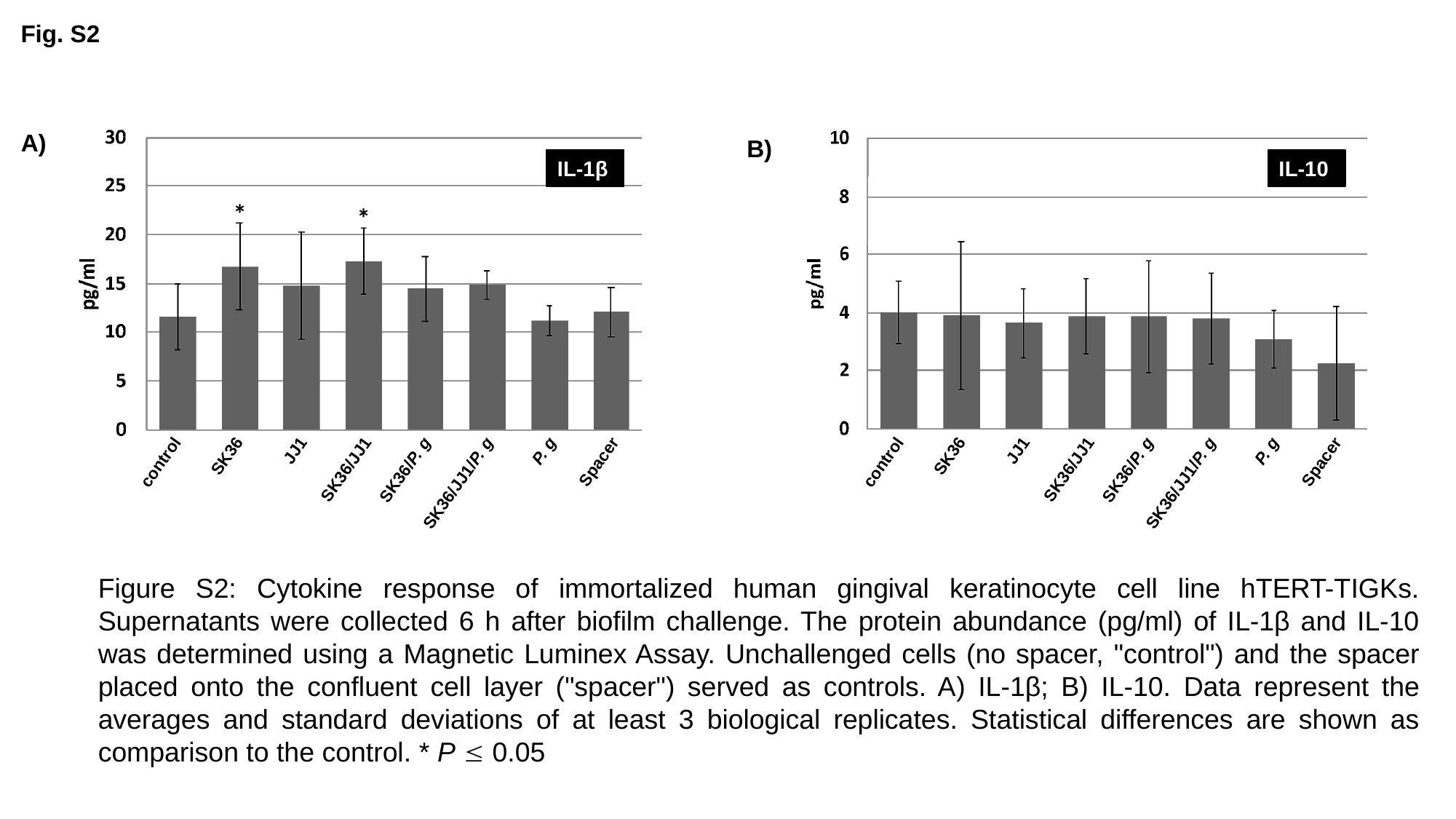

Fig. S2
A)
B)
IL-1β
IL-10
*
*
P. g
JJ1
SK36
Spacer
control
SK36/JJ1
SK36/P. g
SK36/JJ1/P. g
P. g
JJ1
SK36
Spacer
control
SK36/JJ1
SK36/P. g
SK36/JJ1/P. g
Figure S2: Cytokine response of immortalized human gingival keratinocyte cell line hTERT-TIGKs. Supernatants were collected 6 h after biofilm challenge. The protein abundance (pg/ml) of IL-1β and IL-10 was determined using a Magnetic Luminex Assay. Unchallenged cells (no spacer, "control") and the spacer placed onto the confluent cell layer ("spacer") served as controls. A) IL-1β; B) IL-10. Data represent the averages and standard deviations of at least 3 biological replicates. Statistical differences are shown as comparison to the control. * P ≤ 0.05

## Slide 4
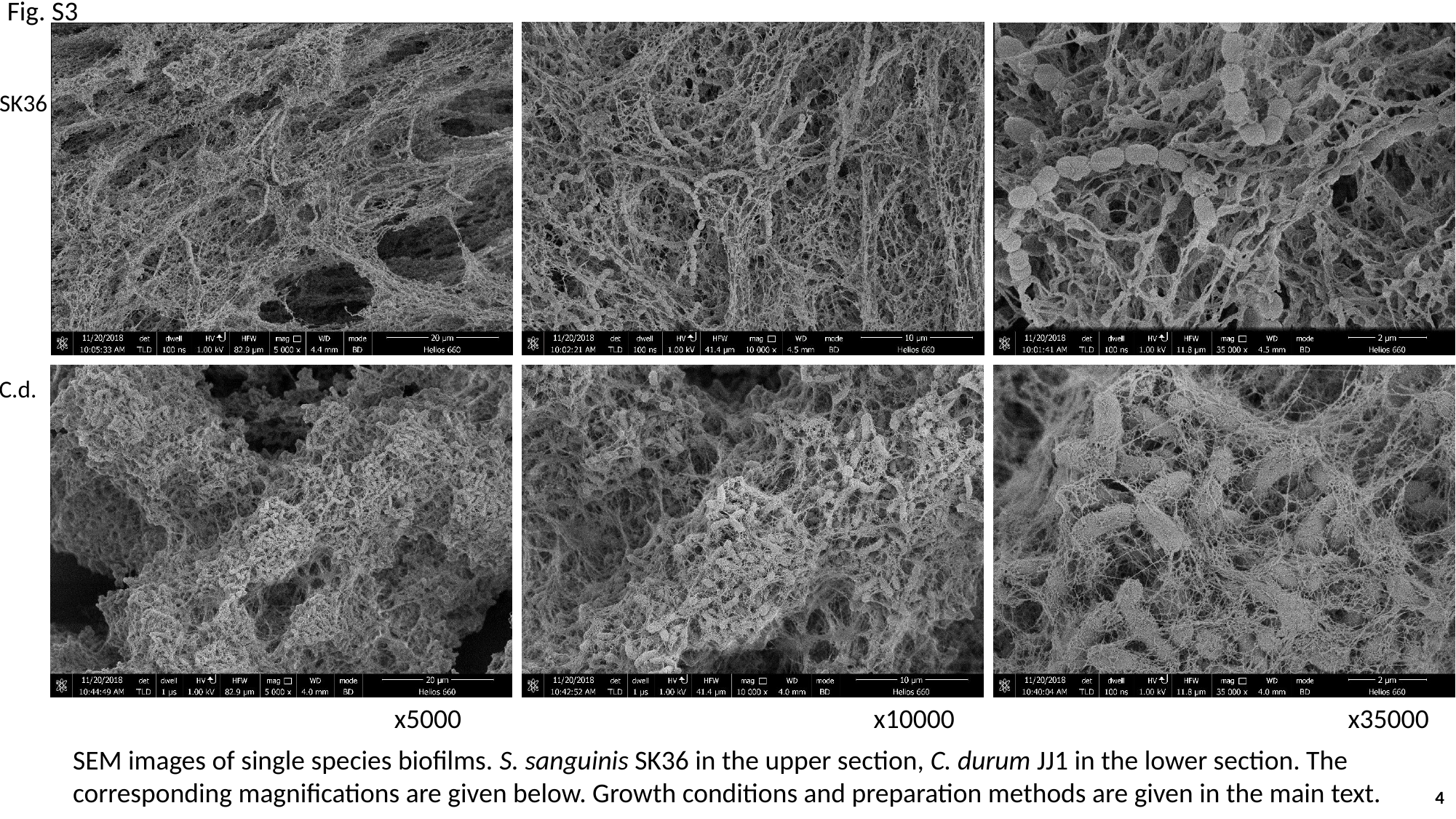

Fig. S3
SK36
C.d.
x5000
x10000
x35000
SEM images of single species biofilms. S. sanguinis SK36 in the upper section, C. durum JJ1 in the lower section. The corresponding magnifications are given below. Growth conditions and preparation methods are given in the main text.
4

## Slide 5
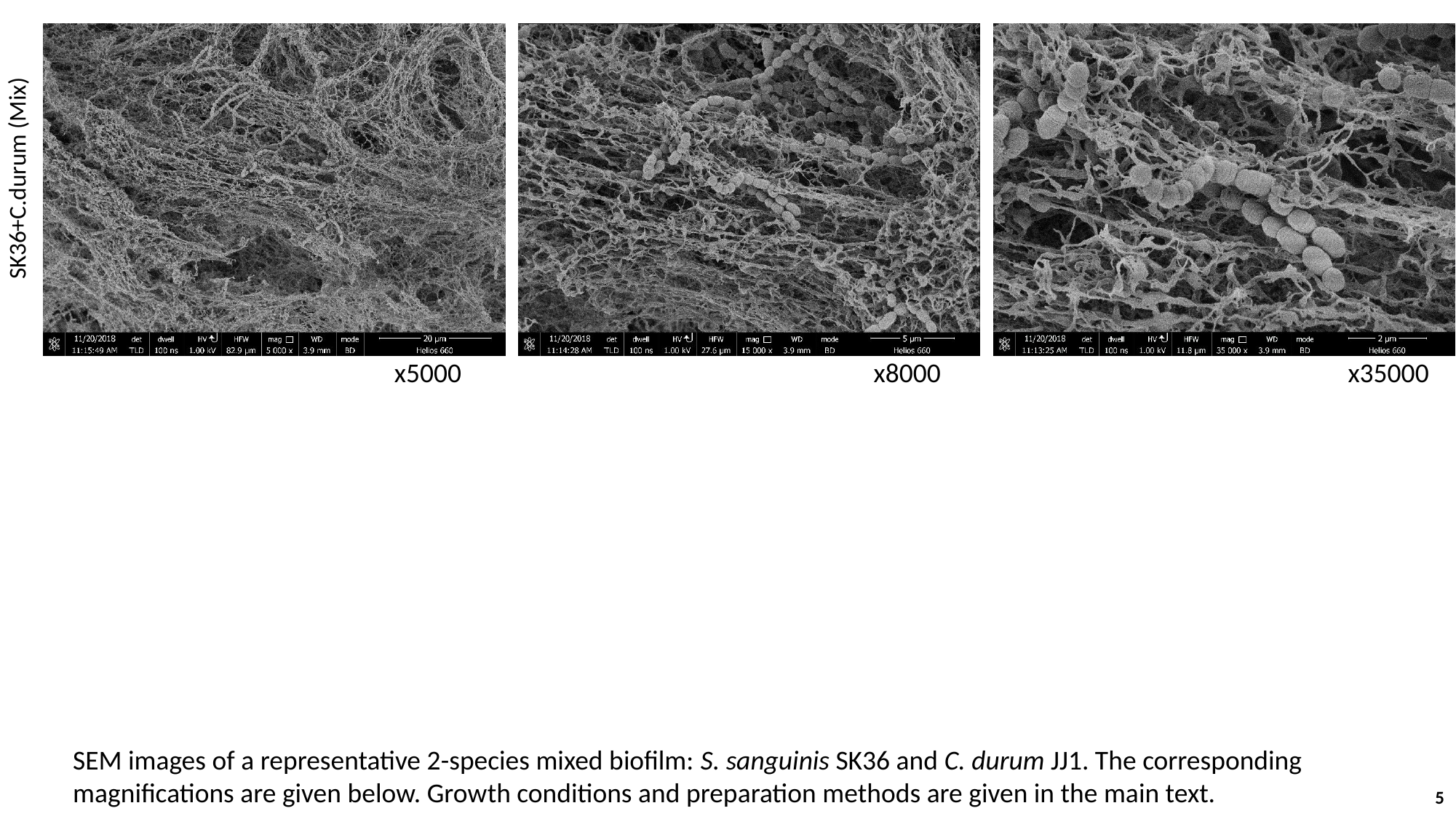

SK36+C.durum (Mix)
x5000
x8000
x35000
SEM images of a representative 2-species mixed biofilm: S. sanguinis SK36 and C. durum JJ1. The corresponding magnifications are given below. Growth conditions and preparation methods are given in the main text.
5

## Slide 6
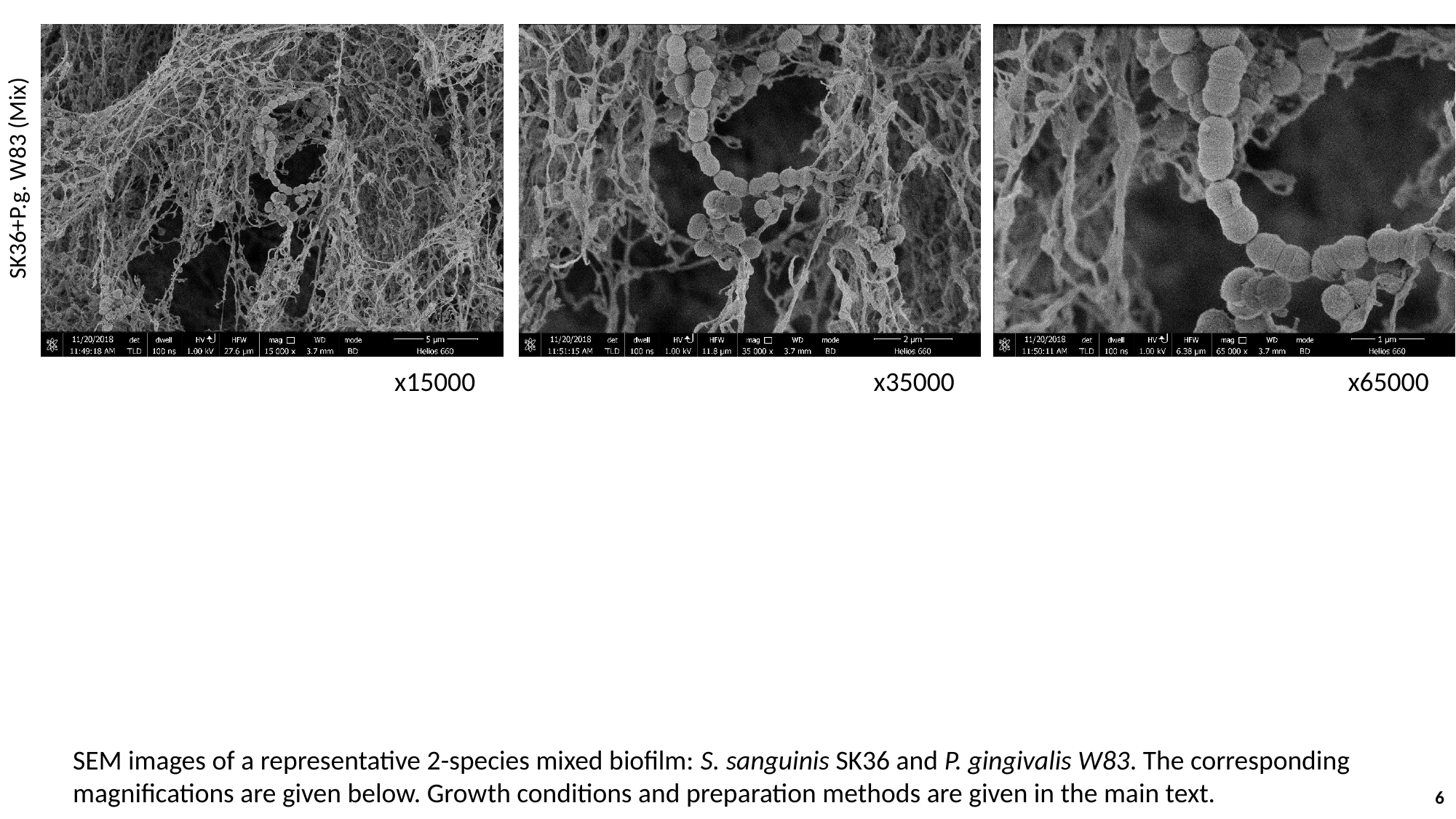

SK36+P.g. W83 (Mix)
x15000
x35000
x65000
SEM images of a representative 2-species mixed biofilm: S. sanguinis SK36 and P. gingivalis W83. The corresponding magnifications are given below. Growth conditions and preparation methods are given in the main text.
6

## Slide 7
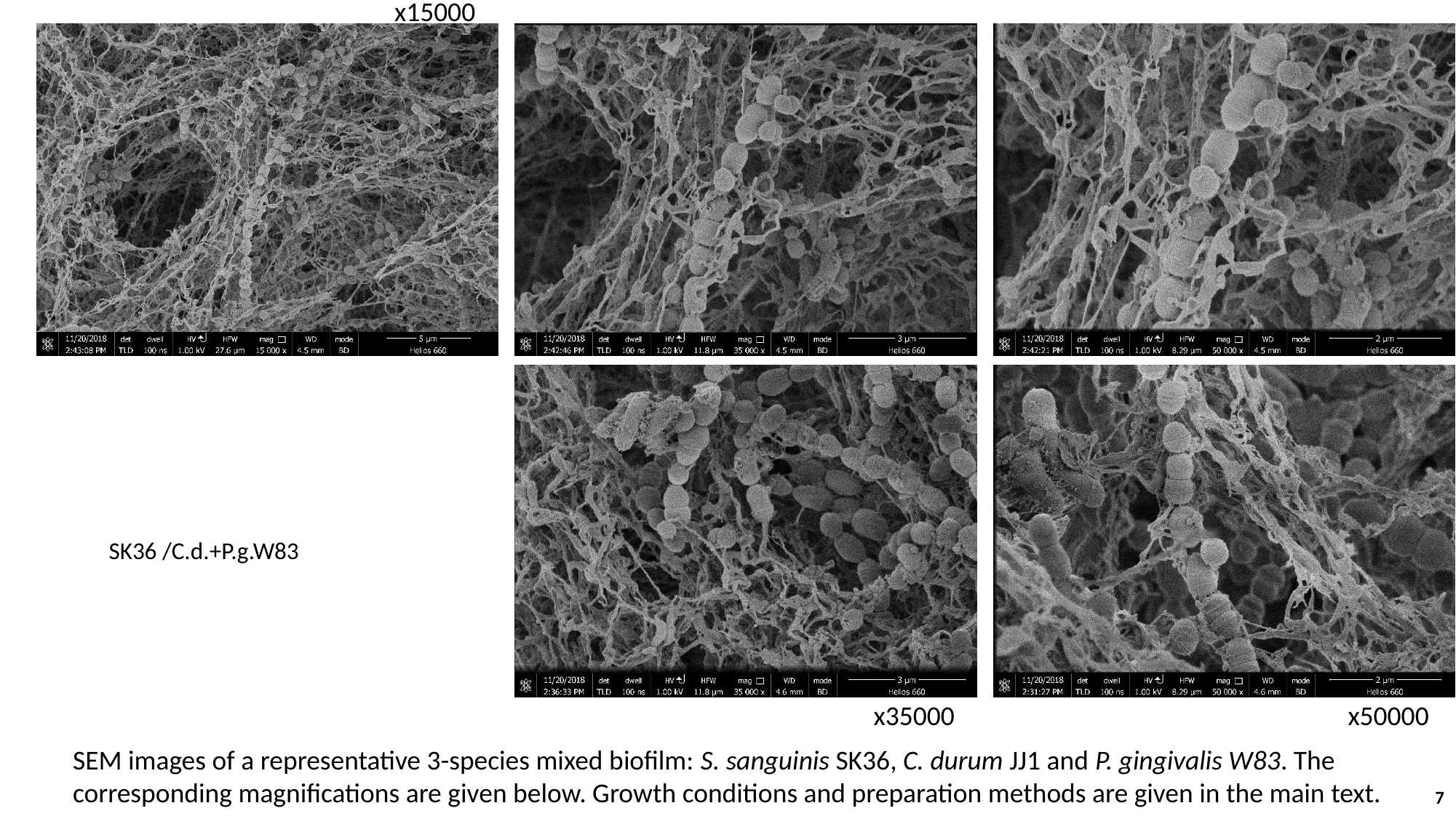

x15000
SK36 /C.d.+P.g.W83
x35000
x50000
SEM images of a representative 3-species mixed biofilm: S. sanguinis SK36, C. durum JJ1 and P. gingivalis W83. The corresponding magnifications are given below. Growth conditions and preparation methods are given in the main text.
7

## Slide 8
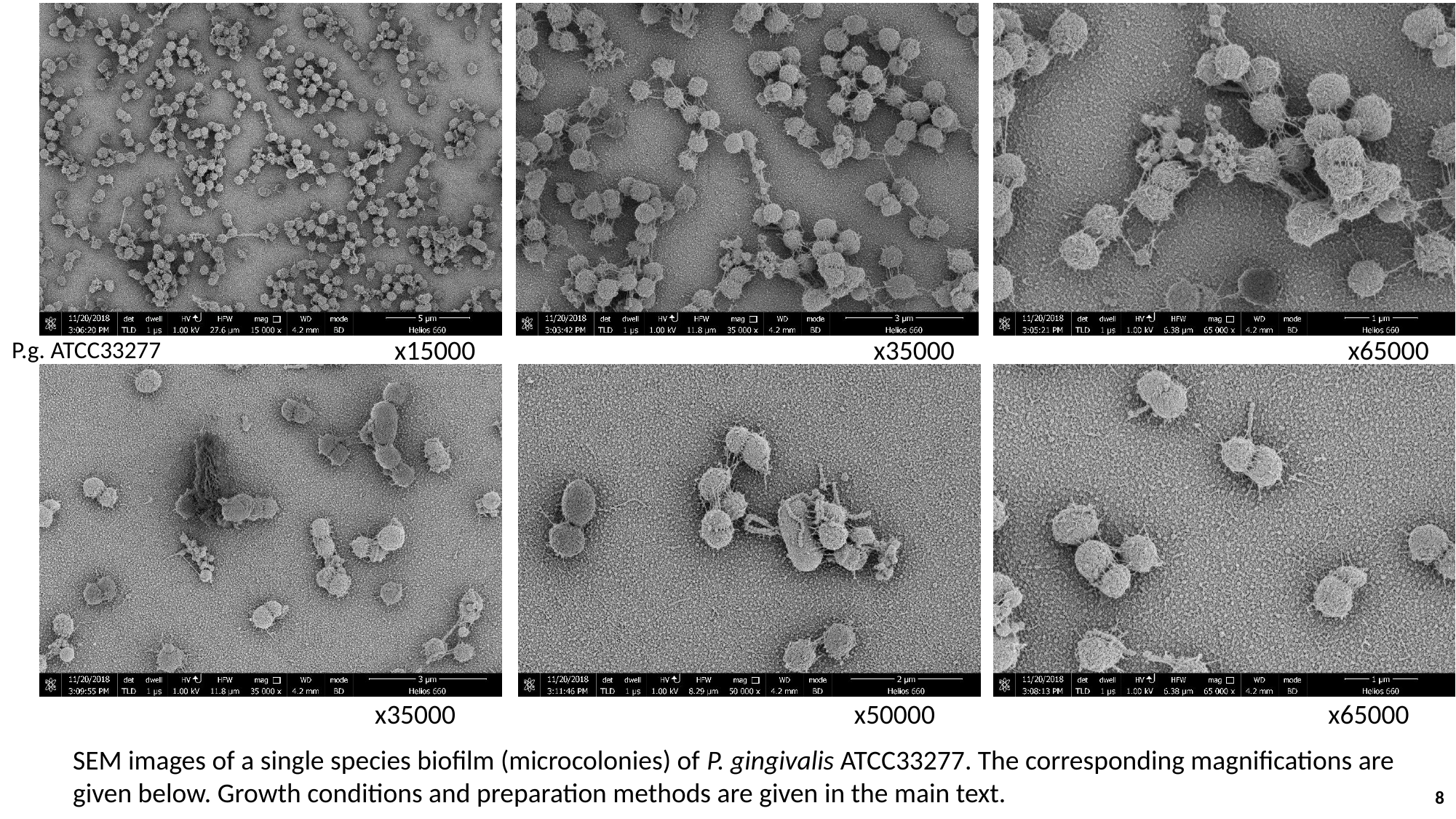

x15000
x35000
x65000
P.g. ATCC33277
x35000
x50000
x65000
SEM images of a single species biofilm (microcolonies) of P. gingivalis ATCC33277. The corresponding magnifications are given below. Growth conditions and preparation methods are given in the main text.
8

## Slide 9
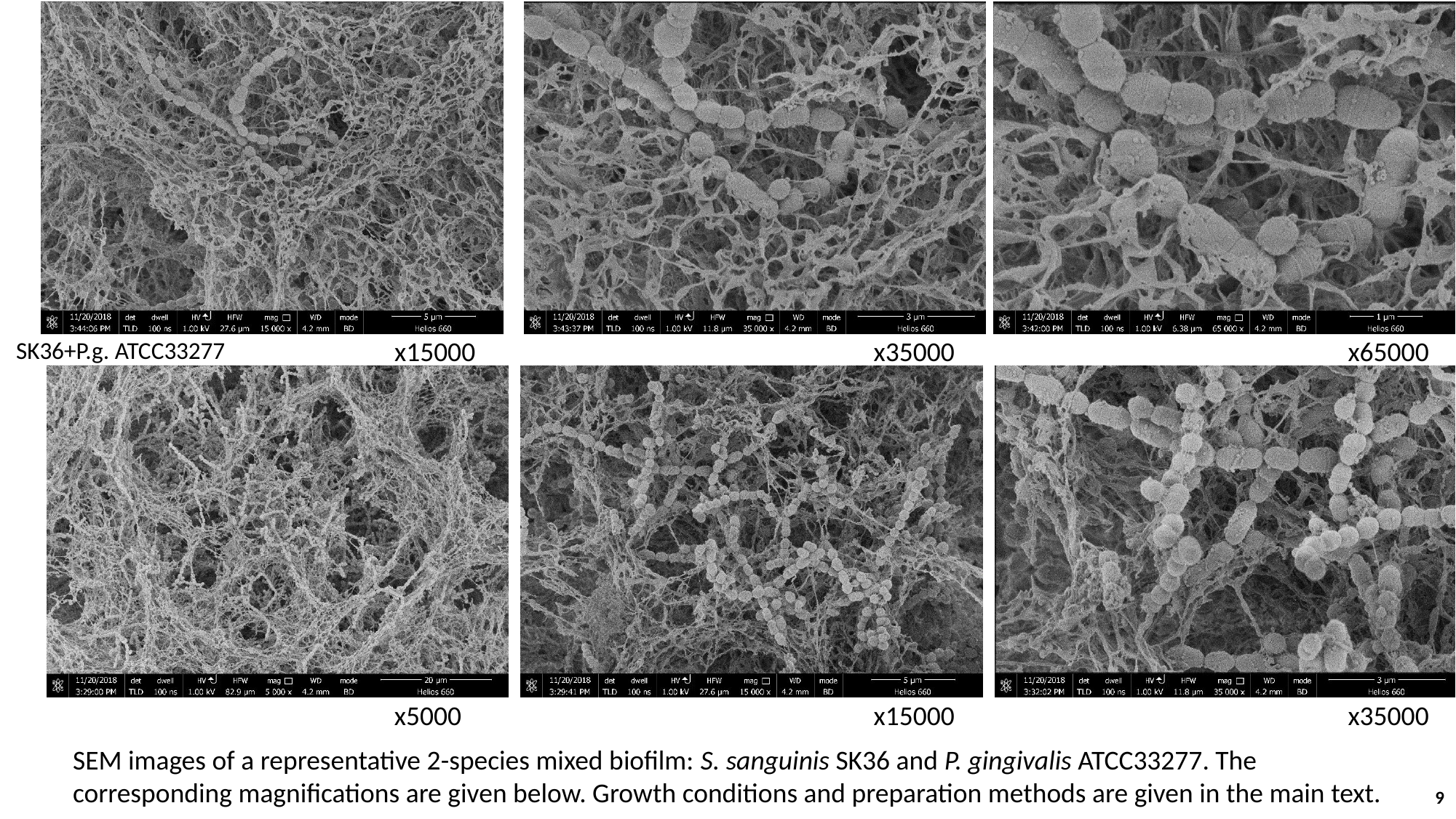

x15000
x35000
x65000
SK36+P.g. ATCC33277
x5000
x15000
x35000
SEM images of a representative 2-species mixed biofilm: S. sanguinis SK36 and P. gingivalis ATCC33277. The corresponding magnifications are given below. Growth conditions and preparation methods are given in the main text.
9

## Slide 10
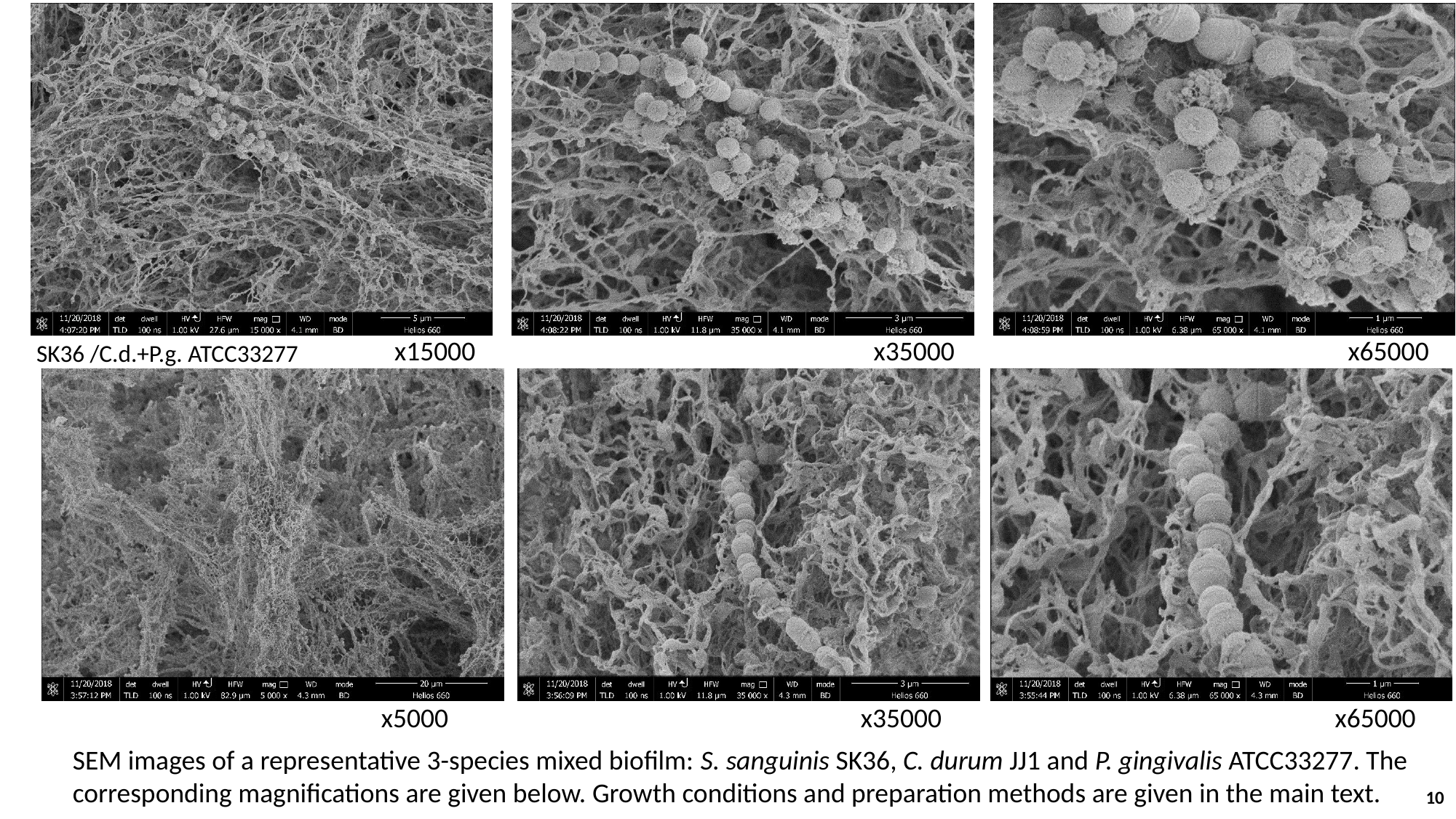

x15000
x35000
x65000
SK36 /C.d.+P.g. ATCC33277
x5000
x35000
x65000
SEM images of a representative 3-species mixed biofilm: S. sanguinis SK36, C. durum JJ1 and P. gingivalis ATCC33277. The corresponding magnifications are given below. Growth conditions and preparation methods are given in the main text.
10

## Slide 11
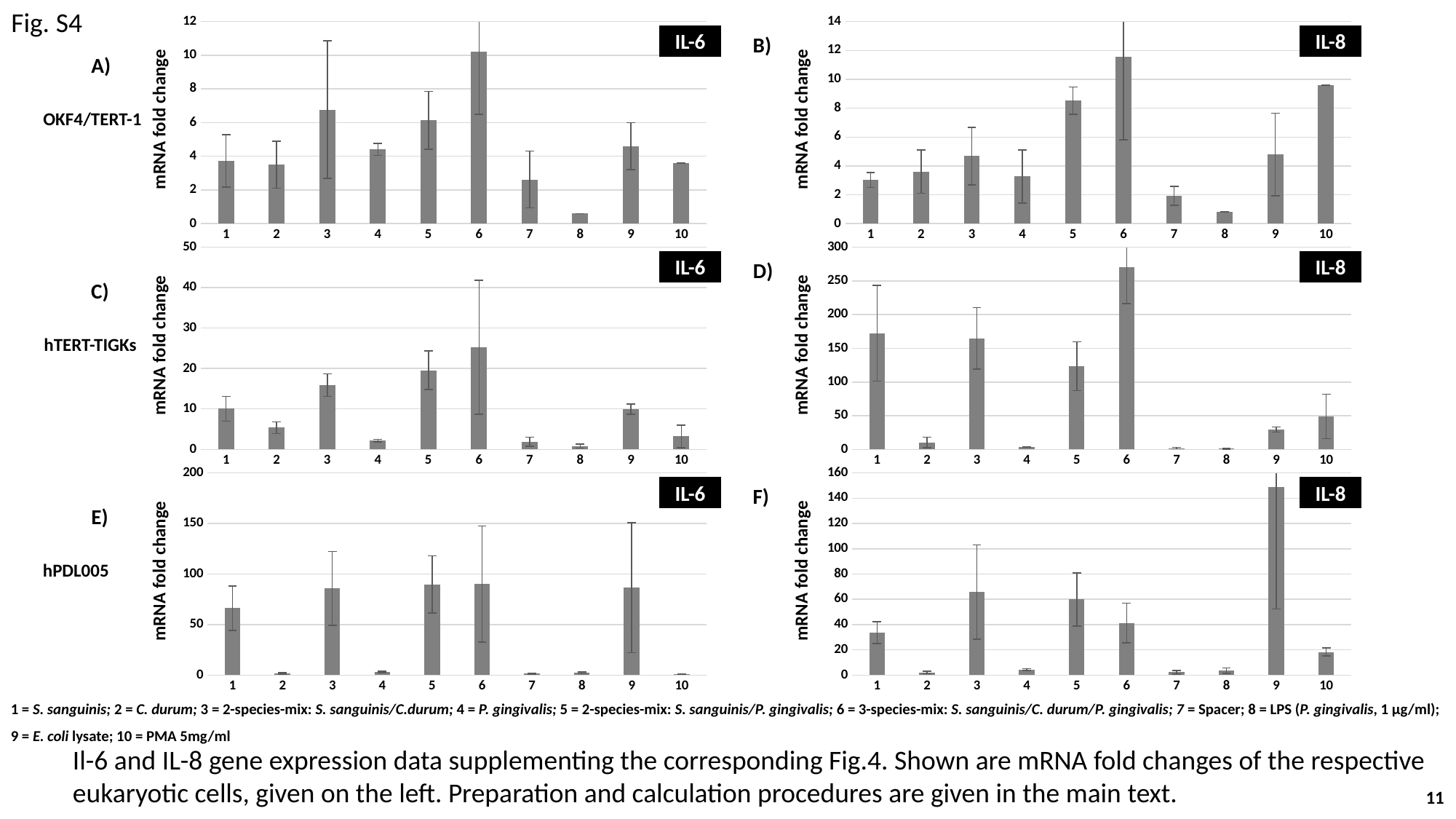

Fig. S4
### Chart
| Category | |
|---|---|
### Chart
| Category | |
|---|---|IL-6
B)
IL-8
A)
OKF4/TERT-1
mRNA fold change
mRNA fold change
### Chart
| Category | |
|---|---|
### Chart
| Category | |
|---|---|IL-6
D)
IL-8
C)
hTERT-TIGKs
mRNA fold change
mRNA fold change
### Chart
| Category | |
|---|---|
### Chart
| Category | |
|---|---|IL-6
F)
IL-8
E)
hPDL005
mRNA fold change
mRNA fold change
1 = S. sanguinis; 2 = C. durum; 3 = 2-species-mix: S. sanguinis/C.durum; 4 = P. gingivalis; 5 = 2-species-mix: S. sanguinis/P. gingivalis; 6 = 3-species-mix: S. sanguinis/C. durum/P. gingivalis; 7 = Spacer; 8 = LPS (P. gingivalis, 1 µg/ml); 9 = E. coli lysate; 10 = PMA 5mg/ml
Il-6 and IL-8 gene expression data supplementing the corresponding Fig.4. Shown are mRNA fold changes of the respective eukaryotic cells, given on the left. Preparation and calculation procedures are given in the main text.
11

## Slide 12
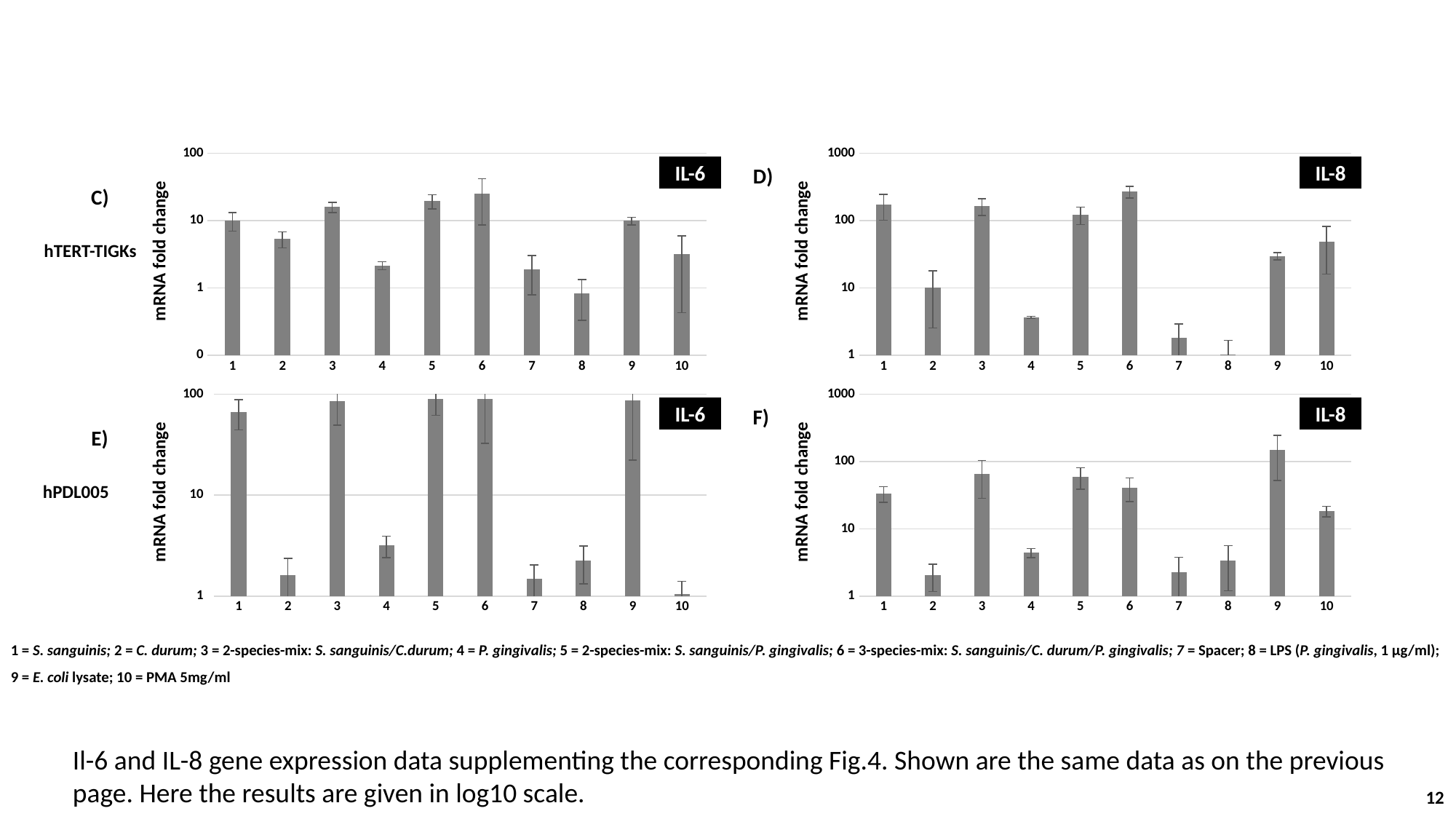

### Chart
| Category | |
|---|---|
### Chart
| Category | |
|---|---|IL-6
D)
IL-8
C)
hTERT-TIGKs
mRNA fold change
mRNA fold change
### Chart
| Category | |
|---|---|
### Chart
| Category | |
|---|---|IL-6
F)
IL-8
E)
hPDL005
mRNA fold change
mRNA fold change
1 = S. sanguinis; 2 = C. durum; 3 = 2-species-mix: S. sanguinis/C.durum; 4 = P. gingivalis; 5 = 2-species-mix: S. sanguinis/P. gingivalis; 6 = 3-species-mix: S. sanguinis/C. durum/P. gingivalis; 7 = Spacer; 8 = LPS (P. gingivalis, 1 µg/ml); 9 = E. coli lysate; 10 = PMA 5mg/ml
Il-6 and IL-8 gene expression data supplementing the corresponding Fig.4. Shown are the same data as on the previous page. Here the results are given in log10 scale.
12

## Slide 13
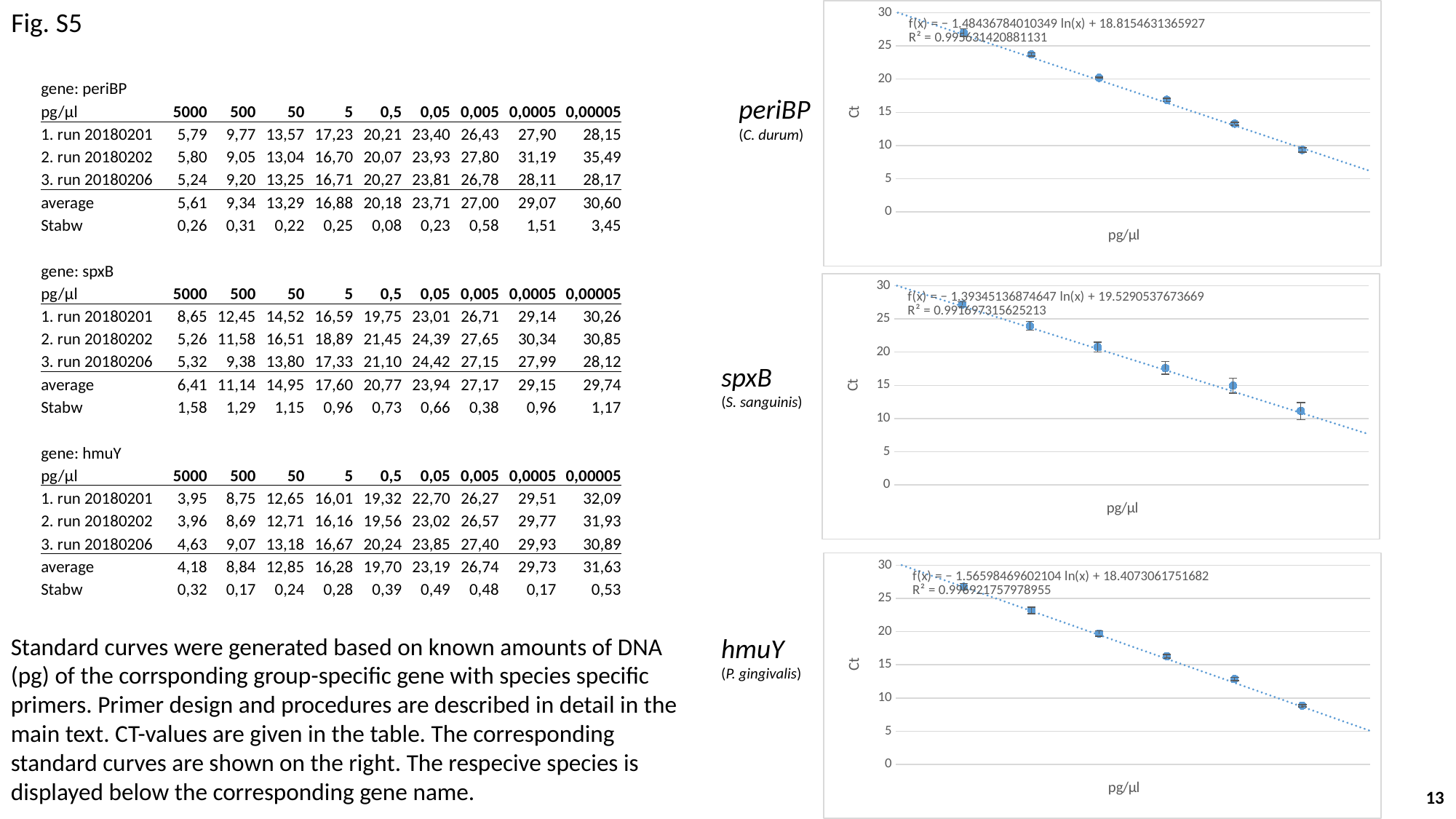

Fig. S5
### Chart
| Category | PeriBP |
|---|---|| gene: periBP | | | | | | | | | |
| --- | --- | --- | --- | --- | --- | --- | --- | --- | --- |
| pg/µl | 5000 | 500 | 50 | 5 | 0,5 | 0,05 | 0,005 | 0,0005 | 0,00005 |
| 1. run 20180201 | 5,79 | 9,77 | 13,57 | 17,23 | 20,21 | 23,40 | 26,43 | 27,90 | 28,15 |
| 2. run 20180202 | 5,80 | 9,05 | 13,04 | 16,70 | 20,07 | 23,93 | 27,80 | 31,19 | 35,49 |
| 3. run 20180206 | 5,24 | 9,20 | 13,25 | 16,71 | 20,27 | 23,81 | 26,78 | 28,11 | 28,17 |
| average | 5,61 | 9,34 | 13,29 | 16,88 | 20,18 | 23,71 | 27,00 | 29,07 | 30,60 |
| Stabw | 0,26 | 0,31 | 0,22 | 0,25 | 0,08 | 0,23 | 0,58 | 1,51 | 3,45 |
| | | | | | | | | | |
| gene: spxB | | | | | | | | | |
| pg/µl | 5000 | 500 | 50 | 5 | 0,5 | 0,05 | 0,005 | 0,0005 | 0,00005 |
| 1. run 20180201 | 8,65 | 12,45 | 14,52 | 16,59 | 19,75 | 23,01 | 26,71 | 29,14 | 30,26 |
| 2. run 20180202 | 5,26 | 11,58 | 16,51 | 18,89 | 21,45 | 24,39 | 27,65 | 30,34 | 30,85 |
| 3. run 20180206 | 5,32 | 9,38 | 13,80 | 17,33 | 21,10 | 24,42 | 27,15 | 27,99 | 28,12 |
| average | 6,41 | 11,14 | 14,95 | 17,60 | 20,77 | 23,94 | 27,17 | 29,15 | 29,74 |
| Stabw | 1,58 | 1,29 | 1,15 | 0,96 | 0,73 | 0,66 | 0,38 | 0,96 | 1,17 |
| | | | | | | | | | |
| gene: hmuY | | | | | | | | | |
| pg/µl | 5000 | 500 | 50 | 5 | 0,5 | 0,05 | 0,005 | 0,0005 | 0,00005 |
| 1. run 20180201 | 3,95 | 8,75 | 12,65 | 16,01 | 19,32 | 22,70 | 26,27 | 29,51 | 32,09 |
| 2. run 20180202 | 3,96 | 8,69 | 12,71 | 16,16 | 19,56 | 23,02 | 26,57 | 29,77 | 31,93 |
| 3. run 20180206 | 4,63 | 9,07 | 13,18 | 16,67 | 20,24 | 23,85 | 27,40 | 29,93 | 30,89 |
| average | 4,18 | 8,84 | 12,85 | 16,28 | 19,70 | 23,19 | 26,74 | 29,73 | 31,63 |
| Stabw | 0,32 | 0,17 | 0,24 | 0,28 | 0,39 | 0,49 | 0,48 | 0,17 | 0,53 |
periBP
(C. durum)
### Chart
| Category | SpxB |
|---|---|spxB
(S. sanguinis)
### Chart
| Category | hmuy |
|---|---|Standard curves were generated based on known amounts of DNA (pg) of the corrsponding group-specific gene with species specific primers. Primer design and procedures are described in detail in the main text. CT-values are given in the table. The corresponding standard curves are shown on the right. The respecive species is displayed below the corresponding gene name.
hmuY
(P. gingivalis)
13

## Slide 14
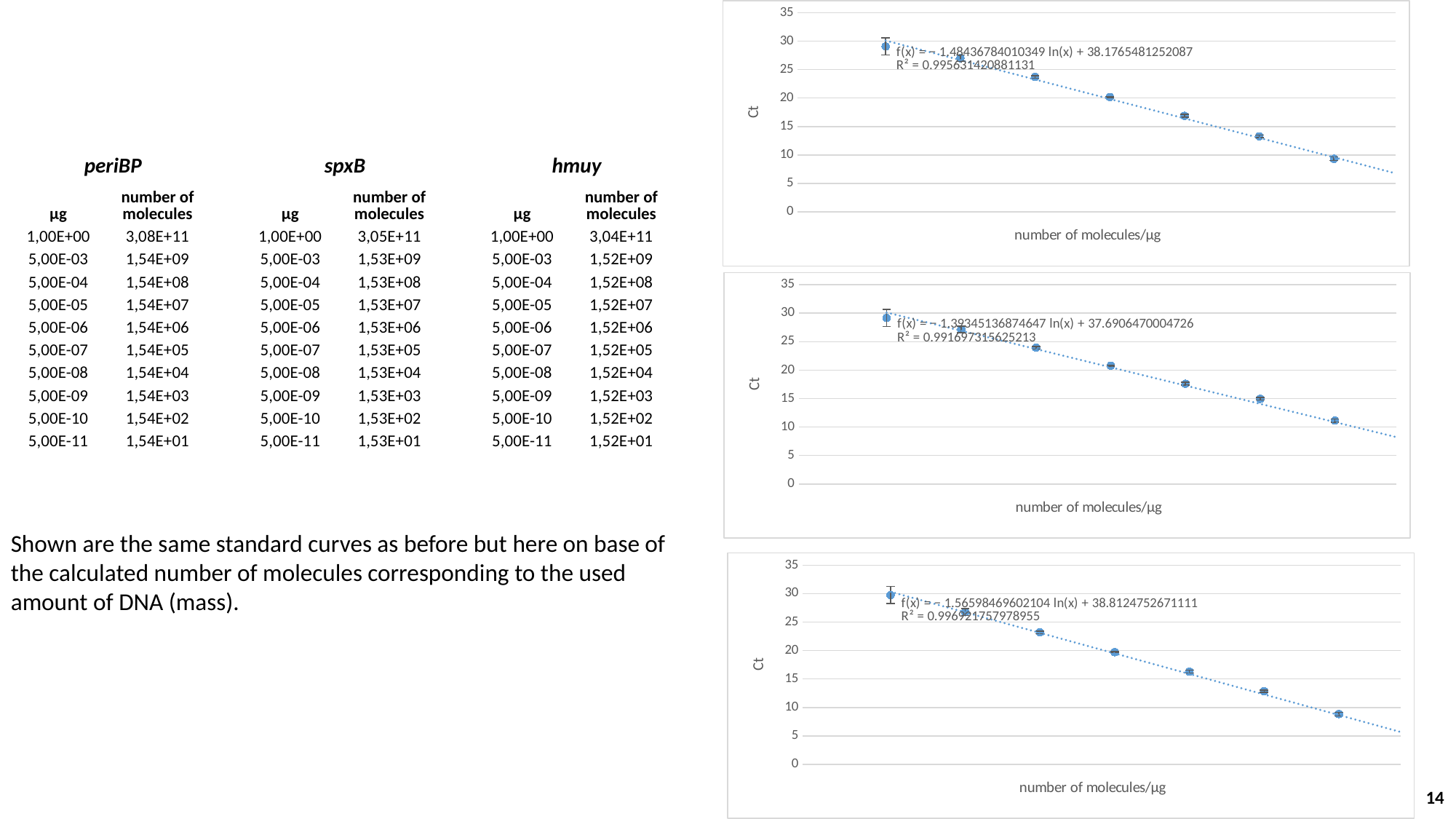

### Chart
| Category | PeriBP |
|---|---|| periBP | | | spxB | | | hmuy | |
| --- | --- | --- | --- | --- | --- | --- | --- |
| µg | number of molecules | | µg | number of molecules | | µg | number of molecules |
| 1,00E+00 | 3,08E+11 | | 1,00E+00 | 3,05E+11 | | 1,00E+00 | 3,04E+11 |
| 5,00E-03 | 1,54E+09 | | 5,00E-03 | 1,53E+09 | | 5,00E-03 | 1,52E+09 |
| 5,00E-04 | 1,54E+08 | | 5,00E-04 | 1,53E+08 | | 5,00E-04 | 1,52E+08 |
| 5,00E-05 | 1,54E+07 | | 5,00E-05 | 1,53E+07 | | 5,00E-05 | 1,52E+07 |
| 5,00E-06 | 1,54E+06 | | 5,00E-06 | 1,53E+06 | | 5,00E-06 | 1,52E+06 |
| 5,00E-07 | 1,54E+05 | | 5,00E-07 | 1,53E+05 | | 5,00E-07 | 1,52E+05 |
| 5,00E-08 | 1,54E+04 | | 5,00E-08 | 1,53E+04 | | 5,00E-08 | 1,52E+04 |
| 5,00E-09 | 1,54E+03 | | 5,00E-09 | 1,53E+03 | | 5,00E-09 | 1,52E+03 |
| 5,00E-10 | 1,54E+02 | | 5,00E-10 | 1,53E+02 | | 5,00E-10 | 1,52E+02 |
| 5,00E-11 | 1,54E+01 | | 5,00E-11 | 1,53E+01 | | 5,00E-11 | 1,52E+01 |
### Chart
| Category | SpxB |
|---|---|Shown are the same standard curves as before but here on base of the calculated number of molecules corresponding to the used amount of DNA (mass).
### Chart
| Category | hmuy |
|---|---|14
